# Roles of the creatine kinase system and myoglobin in maintaining energetic state in the working heart

**DOI:** 10.1186/1752-0509-3-22

**Published:** 2009-02-19

**Authors:** Fan Wu, Daniel A Beard

**Affiliations:** 1 Biotechnology and Bioengineering Center, Department of Physiology, Medical College of Wisconsin, Milwaukee, Wisconsin 53226, USA

## Abstract

**Background:**

The heart is capable of maintaining contractile function despite a transient decrease in blood flow and increase in cardiac ATP demand during systole. This study analyzes a previously developed model of cardiac energetics and oxygen transport to understand the roles of the creatine kinase system and myoglobin in maintaining the ATP hydrolysis potential during beat-to-beat transient changes in blood flow and ATP hydrolysis rate.

**Results:**

The theoretical investigation demonstrates that elimination of myoglobin only slightly increases the predicted range of oscillation of cardiac oxygenation level during beat-to-beat transients in blood flow and ATP utilization. In silico elimination of myoglobin has almost no impact on the cytoplasmic ATP hydrolysis potential (Δ*G*_ATPase_). In contrast, disabling the creatine kinase system results in considerable oscillations of cytoplasmic ADP and ATP levels and seriously deteriorates the stability of Δ*G*_ATPase _in the beating heart.

**Conclusion:**

The CK system stabilizes Δ*G*_ATPase _by both buffering ATP and ADP concentrations and enhancing the feedback signal of inorganic phosphate in regulating mitochondrial oxidative phosphorylation.

## Background

The working heart relies on uninterrupted supplies of oxygen and substrates to maintain its normal function under different workloads [1,2]. However, during the systole the heart muscle contracts and the coronary blood flow is greatly reduced; while during the diastole the heart muscle relaxes, and the coronary blood flow approaches a maximum [1,2].

Abundant myoglobin and creatine pools exist in cardiac tissue, and their roles have been extensively studied [3-8].

The O_2_-Mb binding reaction is

(1) $$Mb+O_{2}⇌MbO_{2}$$

and the creatine kinase reaction is

(2) ADP+CrP⇌ATP+Cr

Myoglobin may work as an oxygen buffer [6,9], facilitate oxygen diffusion at low cellular oxygen tension [10-12], and/or catalyze chemical reactions (such as NO scavenging) [13-15]. Similarly, the creatine kinase system may either buffer cellular ATP levels or facilitate ATP diffusion inside myocytes [7,8,16].

Under normoxic conditions facilitated diffusion of oxygen by oxy-myoglobin is not expected to play a significant role in oxygen transport in the myocardium [6,17,18]. Therefore we consider only the oxygen storage function of myoglobin in this computational study. The importance of CK-facilitated high-energy phosphate transport depends on diffusion path length and diffusivity of ATP and ADP in cardiomyocytes [7]. Since the myofibrils generally have small diameters (1–2 μm) and are surrounded by dense mitochondria, it is possible that CK-facilitated transport does not play a significant role in vivo either [7]. The CK-shuttle theory of Saks et al. [8,19,20] requires restricted intracellular diffusivity of ADP, for which there is no unambiguous experimental evidence. In this study the buffering role of the CK system is investigated, and CK-facilitated diffusion of high-energy phosphate is not considered.

Here, the roles of myoglobin and creatine phosphate in buffering the energy state (i.e., ATP hydrolysis potential) in the working heart are investigated using a multi-scale computer model integrating cellular metabolism with oxygen transport in the cardiac tissue [21,22]. The cellular metabolism model was adopted from a recently-published computer model of energetic metabolism of cardiac mitochondria [23]. The metabolic model is integrated with a model of oxygen transport accounting for heterogeneous oxygenation in cardiac tissue [21].

To examine roles of myoglobin and the CK system, simulations were performed for the normal model (control) and models with myoglobin and CK not active. The simulations demonstrate that the effects of myoglobin on metabolite levels and cytoplasmic ATP hydrolysis potential are barely discernible in the working heart. In contrast, the CK system plays an essential role in maintaining the energetic state in the heart.

## Methods

**Figure 1:**
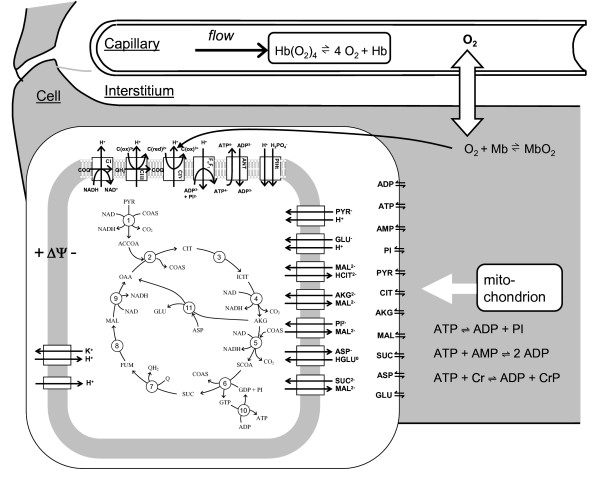
**Diagram of model used to simulate cardiac tissue oxygen transport and energy metabolism**. Oxygen is transported via advection in capillaries, diffuses into cardiomyocytes from capillaries through interstitium, and is reduced into water via the complex IV reaction in mitochondria. Cellular energy metabolism is simulated by a computer model of mitochondrial tricarboxylic acid cycle, oxidative phosphorylation, metabolite transport, and electrophysiology [23].

A computational model previously described and validated [21] is applied to simulate metabolic responses of the beating heart to various workloads. The model components are illustrated in Figure 1. A previously developed and validated axially-distributed oxygen transport models is used to simulate oxygen transport in the cardiac tissue (illustrated in Figure 1). The axially-distributed oxygen transport model is divided into three regions including capillaries, interstitial space, and myocardium, where oxygen binds hemoglobin in red blood cells in capillaries, diffuses into myocardium, and is consumed in mitochondria. A detailed cellular metabolism model is integrated with the multiple-pathway model of oxygen transport, as illustrated in Figure 1. For detailed descriptions of the model, see the Methods and Appendices sections in Wu et al. [21,23]. All model parameters and initial conditions are the same as those used in our previous work [21]. To reduce computational cost, we modify the model described in Reference [21] by reducing the number of the pathways from ten to one and assuming that the ATP hydrolysis rate remains constant during systole. The resulting single-pathway transport model lacks the capability of the multiple-pathway model to characterize the heterogeneous oxygenation in cardiac tissue under ischemia, but still provides a valid and reliable description of oxygenation under normal physiological conditions as simulated in this work [21,22]. The nomenclature and symbols used in this paper are defined in Table 1. Detailed descriptions of the computational model components are included in Additional file 1.

**Table 1:** Nomenclature

**Symbol**	**Definition**	**Units**
*C*	Buffer capacity of the CK system	mol^2 ^kJ^-1^
*C*_Mb_	Myoglobin concentration	μM
CR_tot_	Total creatine pool in myocardium	mmol (l cytoplasm water)^-1^
Δ*G*_ATPase_	Cytoplasmic ATP hydrolysis potential	kJ mol^-1^
$\left|\Delta {\bar{G}}_{\text{ATPase}}\right|$	Average magnitude of cytoplasmic ATP hydrolysis potential	kJ mol^-1^
$\Delta G_{\text{ATPase}}^{\text{o'}}$	Transformed Gibbs free energy of ATP hydrolysis	kJ mol^-1^
ΔΨ	Mitochondrial inner membrane potential	mV
*G*	Blood flow	ml min^-1 ^(g tissue)^-1^
*HR*	Heart rate	beats min^-1^
*I*	Ionic strength	M
*J*_ANT_	Mitochondrial ANT transport flux	mmol sec^-1 ^(liter mito)^-1^
*J*_ATPase_	Cytoplasmic ATP consumption rate	mmol sec^-1 ^(liter cell)^-1^
*MVO*_2_	Oxygen consumption rate	μmol min^-1 ^(g tissue)^-1^
$P_{{\text{O}}_{\text{2}}\text{,cell}}$	Partial oxygen pressure in myocardium	mmHg
*P*_50, Mb_	Half-saturation partial pressure for the oxygen-myoglobin binding	mmHg
*R*	Gas constant	kJ mol^-1 ^K^-1^
*S*_Mb_	Myoglobin saturation	unitless
*T*	Temperature	K
*t*	Time	second

**Figure 2:**
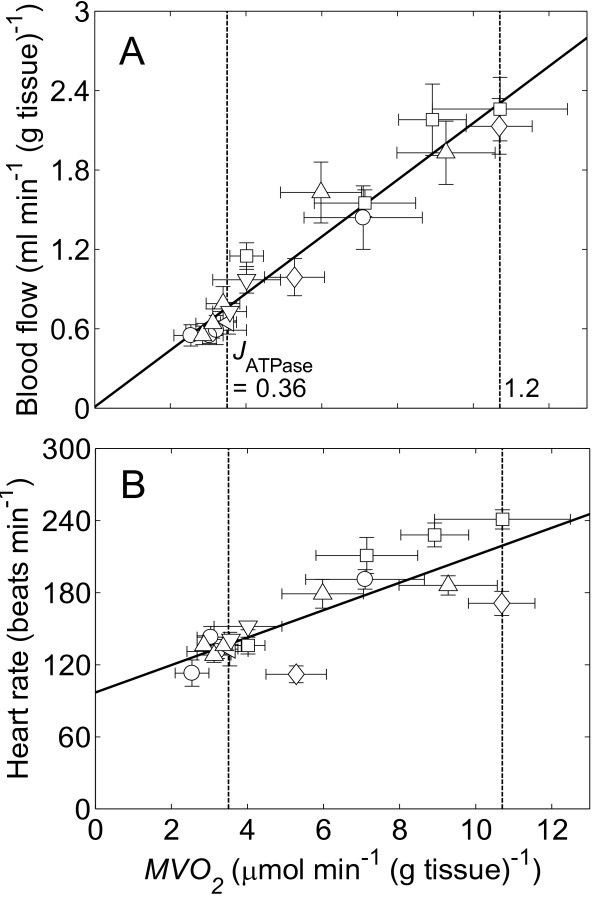
**Relationships between oxygen consumption rate (*MVO*_2_), blood flow (*F*), and heart rates (*HR*) in cardiac tissue**. (A.) Plot of blood flow against oxygen consumption rate. (B.) Plot of heart rate against oxygen consumption rate. Experimental data are obtained from the following sources: ○, Zhang et al. [25]; ◁, Zhang et al. [24]; ◇, Gong et al. [26]; △, Ochiai et al. [27]; ▽, Gong et al. [28]; □, Bache et al. [29]. The relationship between *J*_ATPase_, the ATP hydrolysis rate, and *MVO*_2 _is predicted by the computer model. The solid lines represent the best fits to the data: *G *= 0.2146 *MVO*_2 _+ 0.0093 with R-squared value (R^2^) = 0.9414 (in Figure A) and *HR *= 11.415 *MVO*_2 _+ 96.95 with R^2 ^= 0.6892 (in Figure B). The vertical dashed lines mark baseline (*J*_ATPase _= 0.36 mmol s^-1 ^(l cell)^-1^) and high *MVO*_2 _(*J*_ATPase _= 1.2 mmol s^-1 ^(l cell)^-1^). *J*_ATPase _is expressed in units of mmol s^-1 ^(l cell)^-1^, *G *in units of ml min^-1 ^(g tissue)^-1^, and *HR *in units of beats min^-1^.

Data on blood flow and heart rate under different oxygen consumption rates are collected from a series of experimental data on *in vivo *dog hearts reported by Zhang et al. in Figure 2[24-29]. Here measured blood flow and heart rate are shown at a number of measured oxygen consumption rates. At the mean resting conditions baseline work rate (*MVO*_2 _= 3.5 μmol min^-1 ^(g tissue)^-1 ^and *J*_ATPase _= 0.36 mmol sec^-1 ^(liter cell)^-1^) blood flow and heart rate are 0.76 ml min^-1 ^(g tissue)^-1 ^and 137 beats min^-1^, respectively. At the average maximum observed work rate (*MVO*_2 _= 10.7 μmol min^-1 ^(g tissue)^-1 ^and *J*_ATPase _= 1.2 mmol sec^-1 ^(liter cell)^-1^) blood flow and heart rate are 2.31 ml min^-1 ^(g tissue)^-1 ^and 219 beats min^-1^, respectively. The data in Figure 2 are used to determined relationships between rate of oxygen consumption and average blood flow and heart rate. The linear relationships are *G *= 0.2146 *MVO*_2 _+ 0.0093 and *HR *= 11.415 *MVO*_2 _+ 96.95, where *MVO*_2 _is expressed in units of μmol min^-1 ^(g tissue)^-1^, *G *is flow in units of ml min^-1 ^(g tissue)^-1^, and *HR *is heart rate in units of beats min^-1^.

**Figure 3:**
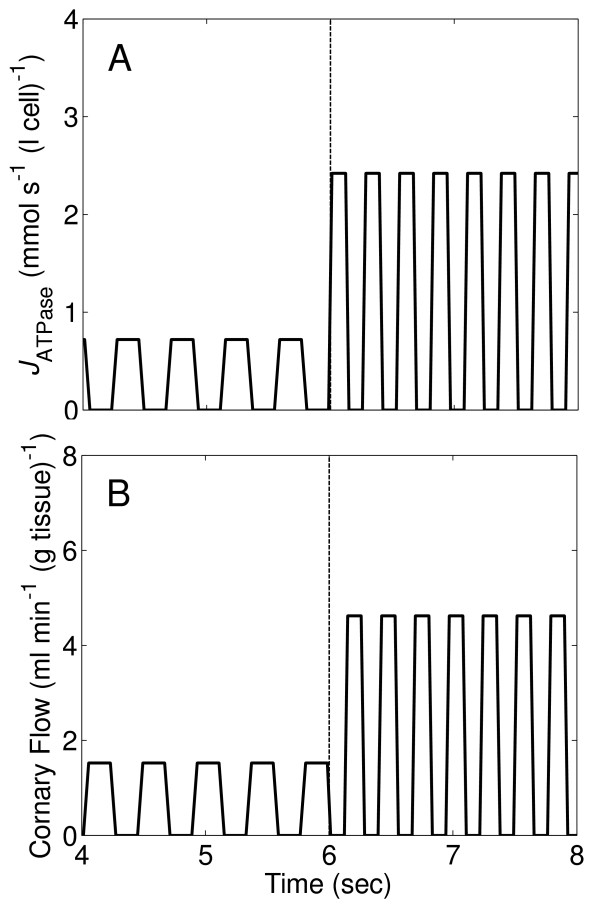
**Temporal profiles of blood flow and cytoplasmic ATP consumption rate used as model inputs**. (A.) Time course of blood flow with a step change occurs at time *t *= 6 seconds corresponding to an increase of cardiac work rate from baseline to maximum level. (B.) Time course of cytoplasmic ATP consumption rate (*J*_ATPase_) with a step change from baseline to maximum work rate at *t *= 6 seconds.

To simulate dynamic blood flow and ATP hydrolysis activity on the beat-to-beat time scale in the working heart, square waves of blood flow and ATPase activity are used, as shown in Figure 3A and 3B. This assumes that blood flow totally stops and ATP consumption rate is nonzero only during systole. Conversely, blood flow is nonzero and ATP consumption is zero during diastole. Experimental observations on coronary blood flow and left ventrical pressure show that neither coronary blood flow nor ATP consumption goes to zero during the heart cycle [30]. Thus the square waves of blood flow and ATP hydrolysis activity shown in Figure 3A and 3B simulate an extreme mismatch between blood flow and ATP consumption. This simplified model allows us to probe a theoretical upper limit on the range of oscillation of concentrations of biochemical reactants.

Oxygen tension in the three compartments (capillaries, interstitial space, and myocardium), mitochondrial membrane potential, and concentrations of metabolites are obtained from model simulations. Myoglobin saturation is computed from oxygen tension in myocardium ($P_{{\text{O}}_{\text{2}}\text{,cell}}$) as

(3) $$S_{\text{Mb}}=\frac{P_{{\text{O}}_{\text{2}}\text{,cell}}}{P_{{\text{O}}_{\text{2}}\text{,cell}}+P_{\text{50,Mb}}},$$

where *P*_50, Mb _is the half-saturation partial pressure for the oxygen-myoglobin binding, 2.39 mmHg [22].

The free energy potential of ATP hydrolysis is computed from

(4) $$\Delta G_{\text{ATPase}}=\Delta G_{\text{ATPase}}^{\text{o'}}+RT\ln\frac{{\text{[ADP]}}_{\text{c}}{\text{[Pi]}}_{\text{c}}}{{\text{[ATP]}}_{\text{c}}},$$

where $\Delta G_{\text{ATPase}}^{\text{o'}}$ = -34.89 kJ mol^-1 ^[31] at ionic strength *I *= 0.17 M, and temperature *T *= 310.15 K, and *R *is the gas constant equal to 8.314 kJ mol^-1 ^K^-1^. The subscript "c" denotes cytoplasm.

Oxygen-myoglobin binding is assumed to be maintained in equilibrium in the tissue model. The kinetics of the CK reaction is governed according to the model described in Reference [21]. Molecular diffusion of creatine, phosphocreatine, and myoglobin are not included in the model (see Reference [21]). Therefore putative roles of oxy-myoglobin and phosphocreatine are not explored in this work.

## Results

To understand the roles of myoglobin and the creatine kinase system as buffers in maintaining the energetic state in the heart, we simulate transient responses and steady states of cardiac energetics under different cardiac workloads in three systems: (1) a normal system with both the normal myoglobin level and creatine kinase activity; (2) a system without myoglobin (no-Mb system); (3) a system with zero creatine kinase activity (no-CK system). The following analyses demonstrate the importance of the CK system in stabilizing energetic states in the beating heart. In the normal system, the myoglobin concentration (*C*_Mb_) is set to be a physiologically reasonable value 200 μM [32], and the creatine kinase activity is set to be an arbitrary large value to ensure that the CK reaction is rapid enough to remain near equilibrium [33]. *C*_Mb _is reduced to zero in the no-Mb system, and the creatine kinase activity is set to be zero in the no-CK system.

### Transient cardiac energetics in responses to a step change of workload

#### The normal system

**Figure 4:**
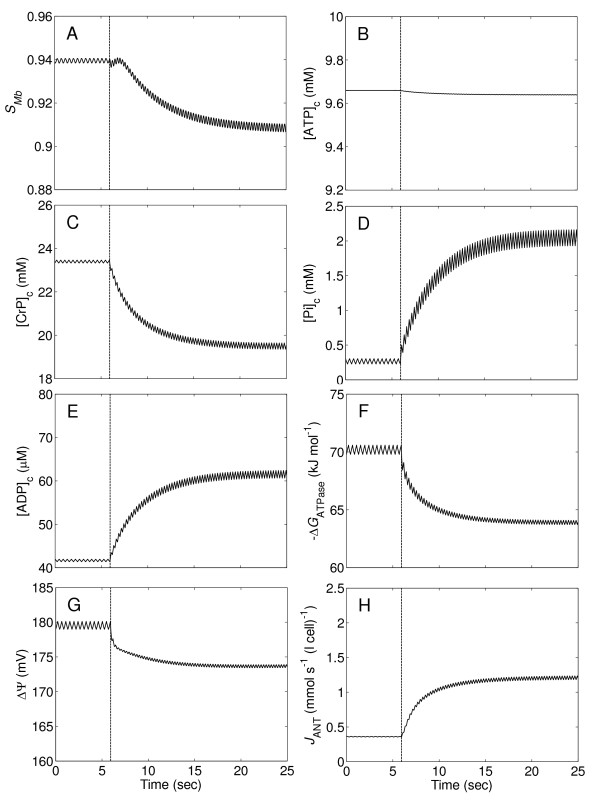
**Transient changes of myoglobin saturation, phosphate levels, cytoplasmic ATP hydrolysis potential, mitochondrial inner membrane potential, and mitochondrial ATP production rate in the control system**. (A.) Myoglobin saturation (*S*_Mb_). (B.) Cytoplasmic ATP concentration ([ATP]_c_). (C.) Cytoplasmic creatine phosphate concentration ([CrP]_c_). (D.) Cytoplasmic inorganic phosphate concentration ([Pi]_c_). (E.) Cytoplasmic ADP concentration ([ADP]_c_). (F.) Cytoplasmic ATP hydrolysis potential (-Δ*G*_ATPase_). (G.) Mitochondrial inner membrane potential (ΔΨ). (H.) Mitochondrial ATP production rate, equal to mitochondrial adenosine nucleotide translocator flux (*J*_ANT_). All variables are simulated for the time courses of the flow and ATP consumption rate in Figure 3.

Figure 4 illustrates transient changes of myoglobin saturation level (*S*_Mb_), phosphate metabolite levels, cytoplasmic ATP hydrolysis potential (Δ*G*_ATPase_), mitochondrial inner membrane potential (ΔΨ), and mitochondrial ANT transport flux (*J*_ANT_), following a step change of cardiac workload and coronary blood flow (shown in Figure 3) in the normal system. The ANT transport flux is the rate at which ATP is transported from the mitochondrial matrix to the cytoplasm in exchange for ADP. When the step change in work rate occurs at time t = 6 seconds, these variables move from their baseline steady state values to approach new steady-state values at the maximal workload after about 20 seconds. Average *S*_Mb _decreases from 0.94 to 0.91, average [ATP]_c _remains almost constant (slightly decreasing from ~9.66 to ~9.64 mM), average [CrP]_c _decreases from ~23.4 to ~19.5 mM, average [Pi]_c _increases from ~0.28 to ~2.1 mM, average [ADP]_c _increases from ~42 to ~62 μm, average -Δ*G*_ATPase _decreases from ~70.2 to ~63.5 kJ mol^-1^, average ΔΨ decreases from ~180 to ~174 mV, and average *J*_ANT _increases from 0.36 to 1.2 mmol s^-1 ^(l cell)^-1^. The range of oscillation of the variables in *S*_Mb_, [ATP]_c_, [CrP]_c_, [Pi]_c_, [ADP]_c_, and *J*_ANT _increases slightly, but interestingly, the oscillations of -Δ*G*_ATPase _and ΔΨ slightly decrease, implying that despite elevated instability of oxygenation and phosphate metabolite levels, energetic stability is slightly increased with increasing work rate.

#### The no-Mb system

**Figure 5:**
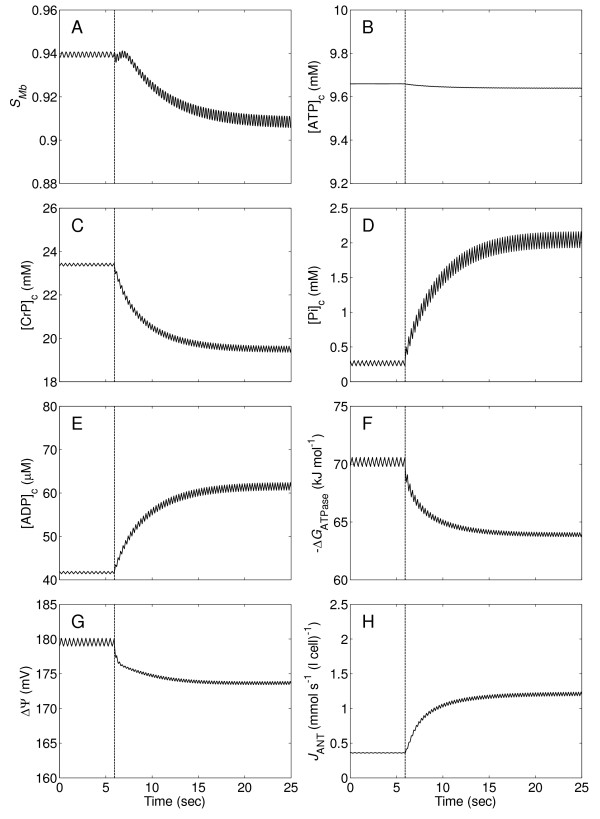
**Transient changes of myoglobin saturation, phosphate levels, cytoplasmic ATP hydrolysis potential, mitochondrial inner membrane potential, and mitochondrial ATP production rate in the no-myoglobin (no-Mb) heart**. As for the control simulation of Figure 4, all variables are simulated for the time courses of the flow and ATP consumption rate in Figure 3.

To investigate the effects of myoglobin on cardiac energetics, simulations of the no-Mb system following the same protocols used above for the normal system were performed. Comparing results from the no-Mb system (shown in Figure 5) with those of the normal system (Figure 4), it is apparent that the myoglobin buffering has little impact on the temporal fluctuations. The oscillations in *S*_Mb _are only slightly higher for the no-Mb system compared to the control. These fluctuations do not impact other energetic variables because the corresponding cellular oxygen tension remains above 15 mmHg at the maximum work rate and does not limit oxidative capacity in the heart [18].

#### The no-CK system

**Figure 6:**
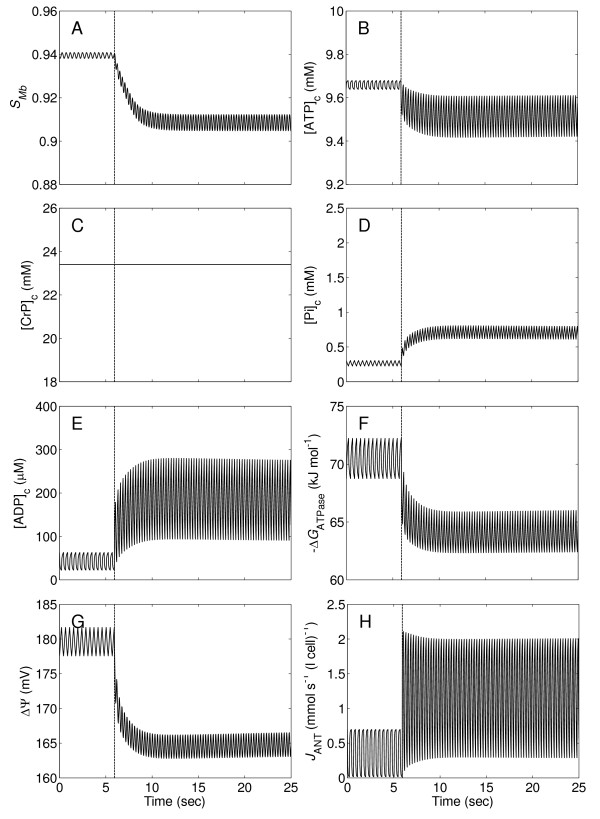
**Transient changes of myoglobin saturation, phosphate levels, cytoplasmic ATP hydrolysis potential, mitochondrial inner membrane potential, and mitochondrial ATP production rate in the heart without creatine kinase activity (no-CK)**. As for the control simulation of Figure 4, all variables are simulated for the time courses of the flow and ATP consumption rate in Figure 3.

To investigate the effects of CK buffering on the system, simulations of the no-CK system were performed using the same protocols used for the control, with the activity of creatine kinase is set to be zero for the simulations. Results illustrated in Figure 6 demonstrate the importance of the CK system in maintaining cardiac energetic state. Compared with the results in Figure 4, disabling the CK system causes considerably enhanced oscillations of *S*_Mb_, [ATP]_c_, [Pi]_c_, [ADP]_c_, Δ*G*_ATPase_, ΔΨ, and *J*_ANT _during the cardiac cycle. In addition, removing CK prevents release of Pi from creatine phosphate (CrP) in response to increases in work rate. As a result, the average [Pi]_c _is reduced from ~2.1 mM in the normal system to ~0.7 mM in the no-CK system at the maximum workload.

#### Steady-state cardiac energetics at varying workloads

**Figure 7:**
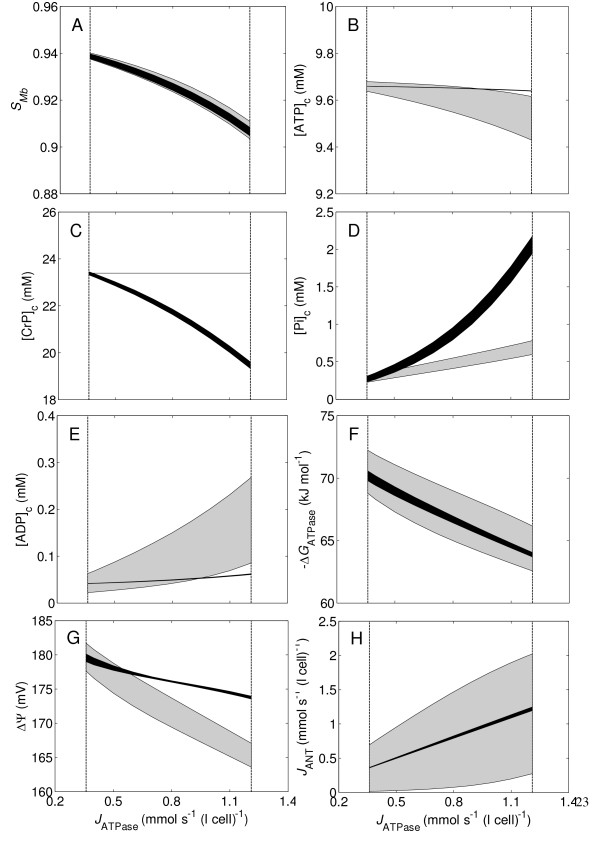
**Steady-state myoglobin saturation, phosphate levels, cytoplasmic ATP hydrolysis potential, mitochondrial inner membrane potential, and mitochondrial ATP production rate in the control and no-CK systems**. Simulated ranges of variables are shown as shaded regions, black for the control and grey for the no-CK system. The upper bounds of the regions represent the maximum values during a heart cycle at steady state, and the low bounds represent the predicted minimum values.

The preceding transient simulations demonstrate the important role of the CK system in maintaining energetic stability in the beating heart. Next, we further explore impacts of the CK system on steady states of *S*_Mb_, [ATP]_c_, [CrP]_c_, [Pi]_c_, [ADP]_c_, Δ*G*_ATPase_, ΔΨ, and *J*_ANT _at varying workloads by simulating steady-state metabolite levels as a function of work rate. In Figure 7, the predicted range (over the cardiac cycle) of each illustrated metabolite is plotted as a shaded region. Black regions represent simulations of the control system; gray represents simulations of the no-CK system. Upper bounds of the regions represent the maximum values of the investigated variables during a heart cycle, and down bounds represent their minimum values.

Figure 7A illustrates that the oxygenation levels in the no-CK system vary slightly more than in the control system, while average oxygenation levels in the both system are nearly identical. Figure 7B shows that [ATP]_c _is maintained at an almost constant level (~9.66 mM) without noticeable oscillations over the whole range of cardiac workload in the control system; in the no-CK system [ATP]_c _oscillates over a wider range (~± 0.2 mM at the maximum workload), and the average [ATP]_c _decreases from ~9.66 mM at the baseline workload to ~9.52 mM at the maximum workload in the no-CK system. Figure 7C shows that the average [CrP]_c _decreases from ~23.4 mM at the baseline workload to ~19.5 mM at the maximum workload in the control system. ([CrP]_c _is constant in the no-CK system because of zero CK activity.) Figure 7D shows that the average [Pi]_c _at the maximal work rate is three-folder lower in the no-CK system than in the control system. Average [Pi]_c _is ~0.7 mM at the maximum workload in the no-CK system compared to ~2.1 mM in the control simulation. Figure 7E shows that [ADP]_c _oscillates drastically in the no-CK system, where [ADP]_c _varies between ~85 μm and ~270 μm at the maximum workload, compared with [ADP]_c _varying between ~60 and ~63 μm at the maximum workload in the control system. As shown in Figure 7F, the average values of Δ*G*_ATPase _are approximately equal between the control and no-CK. However, it is apparent from the predicted ranges of oscillation that the CK system acts in stabilizing Δ*G*_ATPase _in the myocardium. Over the whole range of cardiac workload, the range of oscillations of Δ*G*_ATPase _is approximately four times higher in the no-CK system than in the control. Mitochondrial inner membrane potential (ΔΨ) also oscillates over in a wider range (~± 5 mV) in the no-CK system than in the control system (< ± 1.2 mV) as shown in Figure 7G. Figure 7H shows that in both the control and the no-CK systems the average *J*_ANT _at the steady state is equal to the average cytoplasmic ATP hydrolysis rates at different cardiac workloads, indicating the oxidative phosphorylation flux is able to match the cellular ATP demand in both cases. However, the oxidative ATP synthesis rate in the no-CK case oscillates over a much larger range in the no-CK case.

These results demonstrate two roles of the CK system in maintaining cardiac energetic state. The first role as a buffer has been widely appreciated [7,8,16,34]. The second role is to provide a source of increasing inorganic phosphate with increases in work rate. This allows the cardiomyocytes to maintain relatively stable ATP and ADP concentration, as illustrated in Figure 7B and 7E. Briefly, Figure 7 clearly demonstrates significant roles of the CK system in maintaining energetic stability during the heart cycles at varying cardiac workloads.

## Discussion

### Minor impact of myoglobin on cardiac energetics

Comparisons between Figure 4 and 5 demonstrate the oxygen-storage function of myoglobin plays minor roles in maintaining energy state of the heart in normoxic conditions. However, possible physiological roles of myoglobin may be revealed under extreme conditions. The concentration of myoglobin in the heart of most terrain mammals (e.g., rat, dog, human) is typically between 130 and 320 μM [35,36]. In the event of severe ischemia, these concentrations can only satisfy the oxygen demand of the heart for several seconds even at relatively low work rate. Comparatively, *C*_Mb _level is high in diving birds and mammals (e.g., 4~5 mM in seals) [37], and may contribute to significant oxygen buffering [38].

First hypothesized over 30 years ago, the relative importance of Mb-facilitated oxygen transport has remained unclear, in part, due to conflicting experimental measurements on myoglobin diffusivity [10-12]. Early conclusions on Mb-facilitated oxygen diffusion were mainly drawn based on high estimates (7~23 × 10^-7 ^cm^2 ^s^-1^) of myoglobin diffusivity from in vitro measurements in dilute solution of myoglobin [4,10-12,39,40]. Using two different techniques, microinjection and fluorescence recovery after photobleaching (FRAP), Papadopoulos et al. [9,41] report that that myoglobin diffusivity is approximately 2.0 × 10^-7 ^cm^2 ^s^-1 ^at 37°C in myocardiocytes. Based on this low value of myoglobin diffusivity, Jurgen et al. [6] and Beard and Bassingthwaighte [42] determine that the intracellular myoglobin diffusivity may be too low to provide significant facilitation of oxygen transport in well-oxygenated cardiac tissue. Jue and colleagues [43,44] report higher values of myoglobin diffusion in cardiomyocytes, 7.85 × 10^-7 ^cm^2 ^s^-1 ^at 35°C and 4.24 × 10^-7 ^cm^2 ^s^-1 ^at 22°C, obtained from a ^1^H-NMR technique. However, even at these diffusivity values, myoglobin does not impact oxygen transport into the cells significantly when cellular oxygen tension is above *P*_50 _of myoglobin [43,44]. Similarly, Timmons et al. [45,46] report that oxygen supply does not limit oxidative ATP synthesis in rest-work transition in skeletal muscle, also implying the minor role of myoglobin in oxygen transport in oxidative striated muscle.

Myoglobin can scavenge and catalyze degradation of NO in vivo [13,47]. In addition, Rassaf et al. [14] propose that myoglobin in the heart might work as an oxygen sensor and metabolism regulator, since myoglobin can act as either an NO scavenger at high cellular *P*_O2 _and an NO producer at low *P*_O2_. Since oxygenation in cardiac tissue is maintained above the *P*_50 _for myoglobin in normoxia, myoglobin may act as an NO scavenger under normal conditions.

The absence of myoglobin may elevate cytoplasmic NO level and consequently impacts metabolism regulation, structure, and functions in the heart. Experimental observations show that physiological performance of Mb-knockout mice is comparable to those of normal mice, but the Mb-knockout mice have significantly elevated coronary flow, coronary reserve, and capillary density compared to the control [48,49]. These differences may be associated with elevated NO levels, resulting from reduced NO scavenging in myocardiocytes of the Mb-knockout mice. Elevated NO may also promote angiogenesis by regulating various growth factors and increase capillary density [50-52]. Flogel et al. [53] propose that decreased myoglobin may directly affect substrate selection. For example, elevated NO level impacts substrate utilization by effecting gene expressions of the glucose transporter (GLUT4) and the peroxisome proliferator-activated receptor (PPARα) [53]. Hence, abnormally elevated NO levels may contribute to the physiological adaptations observed in Mb-knockout mice.

In summary, our simulations predict that oxygen buffering by myoglobin in terrestrial mammals plays an insignificant role in maintaining the energy state of the beating heart. Significant changes of structure, hemodynamics, and metabolism observed in Mb-knockout mice [49,53] do not necessarily reflect compensation mechanisms for oxygen deficiency in cardiac tissue. These differences may be caused by elevated cellular NO levels in the knockout mice via various pathways [2,50-56]. In aerobic muscles, such as cardiac muscle, a large amount of mitochondria are required to provide adequate and sustained energy supply, and this high oxidative capacity is usually accompanied by abundant myoglobin, perhaps functioning primarily to scavenge NO generated by mitochondria.

### Creatine kinase system is essential in stabilizing cardiac energetic state

Simulations presented in Figure 6, 7 show considerably diminished energetic stability in the no-CK system, and thus demonstrate significant roles of the creatine kinase system in maintaining normal function in the working heart. These analyses show that the CK system not only buffers cytoplasmic ATP and ADP concentrations but also enhances the Pi feedback signal in regulating mitochondrial oxidative ATP synthesis.

### The CK system enhances the Pi feedback signal in regulating oxidative ATP synthesis

**Figure 8:**
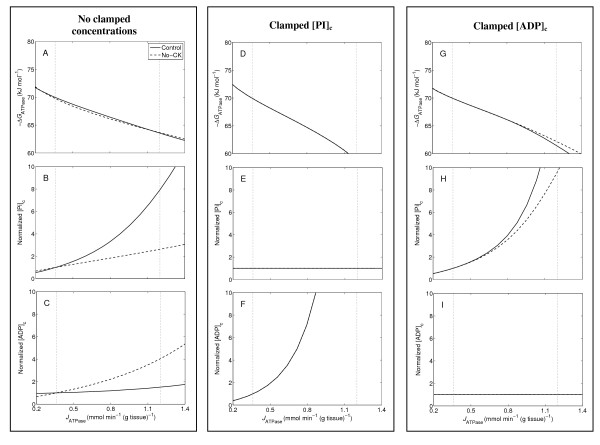
**Steady-state average values of ΔG_ATPase_, [Pi]_c_, [ADP]_c_, and [CrP]_c _plotted against ATP hydrolysis rate (*J*_ATPase_)**. Model-predicted average values of -Δ*G*_ATPase_, normalized [Pi]_c_, and normalized [ADP]_c _are plotted over the range of work rate with no clamped concentrations in A-C, with [Pi]_c _clamped in D-F, and with [ADP]_c _clamped in G-I. The model predictions are plotted as solid lines and dashed lines for the control and no-CK system, respectively. Model-predicted curves of the control and no-CK system overlap in D-F. The baseline and maximum hydrolysis rates, 0.36 and 1.2 mmol s^-1 ^(liter cell)^-1^, are indicated by vertical dotted lines.

Mechanisms underlying regulation of cardiac energetics have been controversial for decades [19,57,58]. Our recently published work suggests that substrate feedback regulation plays an essential role in coordinating mitochondrial ATP synthesis with cellular ATP consumption [21,23,59]. As a product of ATP hydrolysis, inorganic phosphate (Pi) is a primary controller of mitochondrial oxidative ATP synthesis [23,58,60]. As shown in Figure 7 and 8, the concentration of Pi in the no-CK system increases significantly less with work rate in the control system. At the same time ADP increases significantly more in the no-CK system.

One way to understand the relative importance of Pi and ADP in regulating oxidative phosphorylation is to clamp their concentrations and simulate the expected impact on the phosphate metabolites. The concentrations of Pi and ADP are clamped by setting their time derivatives to be zero in the model, respectively. Results from these simulation experiments are illustrated in Figure 8. The model predictions of the control system are plotted as solid lines, and those of the no-CK system are illustrated as dashed lines. The previously obtained baseline and maximum work rates (see Methods section), 0.36 and 1.2 mmol s^-1 ^(liter cell)^-1^, respectively, are indicated by two vertical dotted lines in the plots in Figure 8. The simulations without clamped metabolite concentrations are demonstrated in Figure 8A–C. Figure 8A shows that the magnitudes of average Δ*G*_ATPase _($\left|\Delta {\bar{G}}_{\text{ATPase}}\right|$) are approximately equal between the control and no-CK over the range of workload. Figure 8B illustrates that the average [Pi]_c _only increases approximately two fold over the range of work rate in the no-CK system, compared to the approximately eight-fold increase in the control. Figure 8C shows that the average [ADP]_c _increases approximately four fold over the range of work rate, compared to the only slight increase of the average [ADP]_c _in the control. With [Pi]_c _clamped at the baseline level, the model-predictions are the same in both the control and no-CK system, as shown Figure 8D–F. Figure 8D shows that $\left|\Delta {\bar{G}}_{\text{ATPase}}\right|$ reaches the critical value [21], 63.5 kJ mol^-1^, at *J*_ATPase _= ~0.90 mmol s^-1 ^(liter cell)^-1^. Figure 8F shows a rapid increase of the average [ADP]_c _in responses of elevated work rate. Clamping [ADP]_c _impacts the simulations less significantly, as shown in Figure 8G–I. Figure 8G shows that with [ADP]_c _clampled, $\left|\Delta {\bar{G}}_{\text{ATPase}}\right|$ reaches the critical value at *J*_ATPase _= ~1.04 mmol s^-1 ^(liter cell)^-1 ^in the control, and at *J*_ATPase _= ~1.08 mmol s^-1 ^(liter cell)^-1 ^in the no-CK system. With [ADP]_c _clamped (Figure 8H) and with [ADP]_c _free to vary (Figure 8B), Pi concentrations are higher in the control system than in the no-CK system because of a larger effective total pool of exchangeable phosphates in the control.

To summarize these findings, clamping [Pi]_c _has a more significant impact on energetic stability than clamping [ADP]_c _in both the control and no-CK systems. For both systems, when Pi feedback is removed from the model (by clamping [Pi]_c_) the free energy of ATP hydrolysis drops more quickly with increasing work rate than in the unclamped simulations. When [ADP]_c _is clamped, the impact on the predicted energetic state is less significant than when [Pi]_c _is clamped. Therefore, inorganic phosphate is a more important controller of oxidative phosphorylation than ADP in this model. Since this model is well validated against steady-state and transient in vivo data, it is likely that inorganic phosphate is a primary controller of oxidative phosphorylation in vivo.

Previous analysis of data from purified mitochondria suggests that Pi activates complex III of the respiratory chain [60]. Indeed this phenomenon is incorporated into our computational model [21,23]. When Pi level decreases in the no-CK system, the mitochondrial membrane potential (Figure 6G) is diminished, leading to increased [ADP]_c _and decreased |Δ*G*_ATPase_| compared to control. However, even though the Pi signal is diminished in the no-CK system, Pi remains the most important feedback signal for oxidative phosphorylation.

Saupe et al. [61] observed that as work rate increases in hearts isolated from mice with both mitochondrial and M-form CK knocked out, [Pi]_c _changes little, [ADP]_c _increases about three fold, and $\left|\Delta {\bar{G}}_{\text{ATPase}}\right|$ decreases ~3 kJ mol^-1 ^in these hearts compared to the control. Thus, their concentrations on ADP are qualitatively matched by our simulations, while our model predicts that [Pi]_c _is lower in the no-CK system compared to the control. Since the knockout mouse cardiomyocytes show residual CK activity (40% of wild type), this animal model is not equivalent to our no-CK model. The data of Saupe et al. [61] show a decrease in CrP with increasing work rate, indicating that the CK system is potentially active in the knockout animals.

Feedback of ADP and Pi is also essential for matching oxidative ATP synthesis to cellular energy demand in skeletal muscle [23,59]. However, cytoplasmic Pi can increase to 20 mM and higher at high work rates in skeletal muscle [59] while cytoplasmic Pi is predicted to stay below 3 mM at maximal work rate in the heart [21]. Furthermore, the cytoplasmic Pi concentration in resting slow oxidative soleus muscle is in the range of 5 mM [62]. Therefore even at rest, the Pi concentration is well above the predicted regulatory feedback range for cardiac muscle. We would expect that ADP acts as an important physiological feedback signal in those muscles, as has been established [63,64].

### Analysis of the energy buffering role of the CK system

A simple electrical analog model of Meyer [65] can be used to analyze the buffering role of the CK system in the cardiac energetics. In this model, the buffer capacity of the CK system is computed by the following relationship:

(5) $$\frac{d{\left[\text{CrP}\right]}_{\text{c}}}{d\left|\Delta G_{\text{ATPase}}\right|}=C.$$

**Figure 9:**
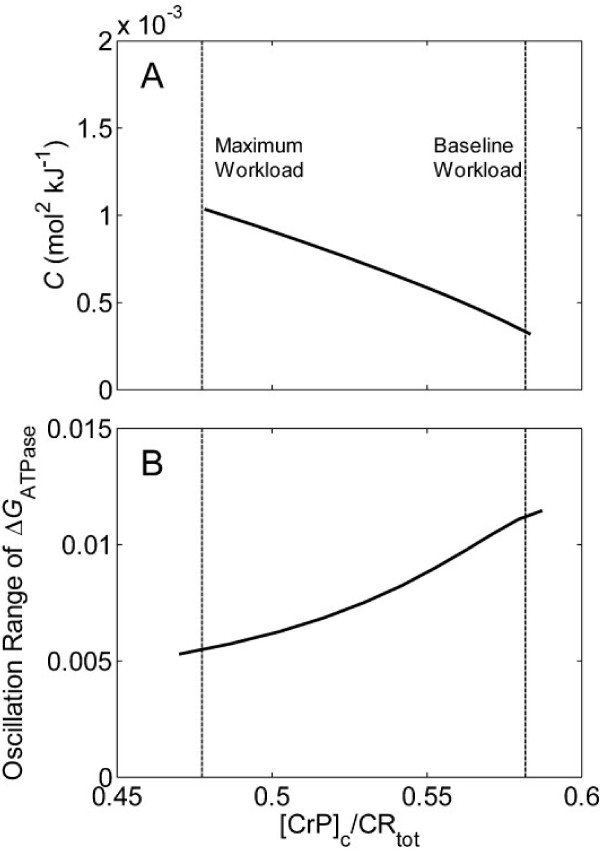
**Buffer capacity of the CK system and range of oscillation of Δ*G*_ATPase _plotted as functions of relative fractions of [CrP]_c_**. (A.) Capacitance of the CK system in buffering Δ*G*_ATPase_, calculated from Equation (5), is plotted against [CrP]_c_/CR_tot _predicted at different work rates. (B.) The predicted range of oscillation of Δ*G*_ATPase _is plotted as $\frac{\max(\left|\Delta G_{\text{ATPase}}\right|)-\min(\left|\Delta G_{\text{ATPase}}\right|)}{\left|\Delta {\bar{G}}_{\text{ATPase}}\right|}$. The curves in A and B are obtained by varying ATP hydrolysis rate from baseline (0.36 mmol s^-1 ^(l cell)^-1^) to maximum (1.2 mmol s^-1 ^(l cell)^-1^) values.

Based on the simulations of [CrP]_c _and Δ*G*_ATPase _at varying cardiac work rates in the normal system (illustrated in Figure 7), Equation (3) can be evaluated based on finite differences. The computed capacitance *C *is plotted Figure 9A against the normalized cytoplasmic CrP ([CrP]_c_/CR_tot_), where CR_tot _is the total creatine pool in myocardium, 40.14 mmol (l cytoplasm water)^-1 ^[66]. As [CrP]_c_/CR_tot _decreases from ~0.58 at the baseline work rate to ~0.48 at the maximum work rate, the value of *C *increases more than three fold (from ~0.33 × 10^-3 ^to ~1.04 × 10^-3 ^mol^2 ^kJ^-1^). As a result, the fluctuations of |Δ*G*_ATPase_| (plotted as $\frac{\max(\left|\Delta G_{\text{ATPase}}\right|)-\min(\left|\Delta G_{\text{ATPase}}\right|)}{\left|\Delta {\bar{G}}_{\text{ATPase}}\right|}$) decreases from ~1.1% to ~0.55%, despite the increased range of oscillations of cytoplasmic ATP consumption rate. In contrast to the almost constant capacitance of the CK system determined for skeletal muscle [65], the CK system is predicted to increase in buffering capacity with work rate in the heart.

### Other possible roles of the CK system

In additional to a temporal buffering function, the CK system has been proposed to facilitate transport of energetic phosphates in myocardium [7,19]. This hypothesis remains controversial. Meyer et al. [7] describe both temporal buffering and "spatial buffering" roles of creatine phosphate associated with the near-equilibrium creatine kinase (CK) reaction and a high cytoplasmic ATP-to-ADP ratio. The spatial buffering role may be negligible in the heart because of small diameters of cardiomyofibrils and abundant surrounding mitochondria [7]. The phosphocreatine shuttle hypothesis – that the free energy of ATP hydrolysis is transported primarily by spatial gradients of CrP and Cr between mitochondria and sites of ATP hydrolysis – hinges on the existence of three critical phenomena: (1) restricted diffusion of adenine nucleotides in cardiomyocytes; (2) functional coupling (direct product-substrate channeling) between mitochondrial adenine nucleotide translocase and creatine kinase; and (3) disequilibrium of the CK reaction in cardiomyocytes [67-70]. While computational models that invoke these three phenomena are able to match data on the kinetics of oxidative phosphorylation in isolated skinned cardiomyocytes [71] and purified mitochondria [69], it remains to be demonstrated that the available data, particularly *in vivo *data, cannot be explained without invoking these phenomena. Indeed, recent measurements of diffusivities of labeled adenine nucleotides in cardiomyocytes demonstrate that bulk diffusion is not restricted to the degree necessary for the phosphocreatine shuttle to operate [72]. The existence and significance of "microcompartments" [69] around mitochondria with restricted diffusion in cardiomyocytes remains an active subject of investigation and debate.

## Conclusion

To determine the roles of myoglobin and the CK system in stabilizing cardiac energy state, a computational model of oxygen transport and cardiac metabolism is applied to simulate transient changes and steady states in the beating heart. The analysis suggests that myoglobin has little impact on cardiac energetics, while the CK system is important for the beating heart to maintain stable energetic state over a range of cardiac work rate. Two distinct functions of the CK system are apparent from this analysis: first, the CK system buffers changes of [ADP]_c _and [ATP]_c _and Δ*G*_ATPase_; second, the CK system enhances the feedback of Pi to match the rate of mitochondrial ATP synthesis to cellular ATP demand while maintaining relatively stable [ADP]_c_.

## Authors' contributions

DAB and FW conceived and designed the experiments, carried out the experiments, and wrote the paper. Both authors read and approved the final manuscript.

## Supplementary Material

Additional file 1 **Supplemental Material for "Roles of the creatine kinase system and myoglobin in maintaining energy state in the working heart".** The supplemental material includes tables listing model components and detailed mathematical descriptions of the computational model. Click here for file
