# Supplementary material for: Roles of the creatine kinase system and myoglobin in maintaining energetic state in the working heart
Source: BMC Syst Biol. 2009 Feb 19;3:22. doi: 10.1186/1752-0509-3-22 (PMC2667476; doi:10.1186/1752-0509-3-22)
Supplement: Additional file 1 — Supplemental Material for "Roles of the creatine kinase system and myoglobin in maintaining energy state in the working heart". The supplemental material includes tables listing model components and detailed mathematical descriptions of the computational model. [file 1752-0509-3-22-S1.doc]

# Supplemental material for “Roles of the creatine kinase system and myoglobin in maintaining energy state in the working heart”

Fan Wu and Daniel A. Beard

Biotechnology and Bioengineering Center, Department of Physiology, Medical College of Wisconsin, Milwaukee, Wisconsin, 53226, United States

Correspondence to: Daniel A. Beard, Medical College of Wisconsin, 8701 Watertown Plank Road, Milwaukee, WI 53226, E-mail:dbeard@mcw.edu, Telephone: (414) 955-5752, Fax: (414) 955-6317.

## S1. List of model components

The computational model applied in the current study integrates computational models of cellular metabolism and oxygen transport in cardiac tissue previously published by Beard et al. [1-3]. Please refer to these published papers and the associated supplemental material for detailed descriptions of model parameterization and validation.

This appendix lists the basic components of the current model. Tables S1 and S2 list the state variables and reaction and transport fluxes considered in the model. Tables S3 lists model parameters.

Table S1: Model variables

| **Variables** | **Descriptions** | **Units** |
| --- | --- | --- |
| Oxygen Concentration: | | |
| C_O2,capillary_ | Total oxygen concentration in capillary | mol (l capillary)^-1^ |
| C_O2,interstitium_ | Total oxygen concentration in interstitium | mol (l interstitium)^-1^ |
| C_O2,cell_ | Total oxygen concentration in myocyte | mol (l cell)^-1^ |
| Mitochondrial membrane potential : | | |
| ΔΨ | Mitochondrial membrane potential | mV |
| Mitochondrial Matrix Variables: | | |
| [H^+^]_x_ | Concentration of H^+^ ion in mito matrix | mol (l matrix water) ^-1^ |
| [K^+^]_x_ | Concentration of K^+^ ion in mito matrix | mol (l matrix water) ^-1^ |
| [Mg^2+^]_x_ | Concentration of Mg^2+^ ion in mito matrix | mol (l matrix water) ^-1^ |
| [NADH]_x_ | Concentration of NADH in mito matrix | mol (l matrix water) ^-1^ |
| [NAD]_x_ | Concentration of NAD in mito matrix | mol (l matrix water) ^-1^ |
| [QH_2_]_x_ | Concentration of reduced ubiquinol in mito matrix | mol (l matrix water) ^-1^ |
| [COQ]_x_ | Concentration of oxidized ubiquinol in mito matrix | mol (l matrix water) ^-1^ |
| [ATP]_x_ | Concentration of total ATP in mito matrix | mol (l matrix water) ^-1^ |
| [ADP]_x_ | Concentration of total ADP in mito matrix | mol (l matrix water)^-1^ |
| [GTP]_x_ | Concentration of total GTP in mito matrix | mol (l matrix water) ^-1^ |
| [GDP]_x_ | Concentration of total GDP in mito matrix | mol (l matrix water)^-1^ |
| [PI]_x_ | Concentration of inorganic phosphate in mito matrix | mol (l matrix water) ^-1^ |
| [PYR]_x_ | Concentration of pyruvate in mito matrix | mol (l matrix water) ^-1^ |
| [COASH]_x_ | Concentration of CoA-SH in mito matrix | mol (l matrix water) ^-1^ |
| [ACCOA]_x_ | Concentration of acetyl-CoA in mito matrix | mol (l matrix water) ^-1^ |
| [OAA]_x_ | Concentration of oxaloacetate in mito matrix | mol (l matrix water) ^-1^ |
| [CIT]_x_ | Concentration of citrate in mito matrix | mol (l matrix water) ^-1^ |
| [ICIT]_x_ | Concentration of isocitrate in mito matrix | mol (l matrix water) ^-1^ |
| [AKG]_x_ | Concentration of α-ketoglutarate in mito matrix | mol (l matrix water) ^-1^ |
| [SCOA]_x_ | Concentration of succinyl-CoA in mito matrix | mol (l matrix water) ^-1^ |
| [SUC]_x_ | Concentration of pyruvate in mito matrix | mol (l matrix water) ^-1^ |
| [FUM]_x_ | Concentration of fumarate in mito matrix | mol (l matrix water) ^-1^ |
| [MAL]_x_ | Concentration of malate in mito matrix | mol (l matrix water) ^-1^ |
| [ASP]_x_ | Concentration of aspartate in mito matrix | mol (l matrix water) ^-1^ |
| [GLU]_x_ | Concentration of glutamate in mito matrix | mol (l matrix water) ^-1^ |
| [O_2_]_x_ | Concentration of oxygen in mito matrix | mol (l matrix water) ^-1^ |
| [CO_2_tot]_x_ | Concentration of total CO_2_ in mito matrix | mol (l matrix water) ^-1^ |
| Mitochondrial Inter-Membrane Space Variables: | | |
| [H^+^]_i_ | Concentration of H^+^ ion in IM space | mol (l IM water) ^-1^ |
| [K^+^]_i_ | Concentration of K^+^ ion in IM space | mol (l IM water) ^-1^ |
| [Mg^2+^]_i_ | Concentration of Mg^2+^ ion in IM space | mol (l IM water) ^-1^ |
| [Cred]_i_ | Concentration of reduced cytochrome C in IM space | mol (l IM water) ^-1^ |
| [Cox]_i_ | Concentration of oxidized cytochrome C in IM space | mol (l IM water) ^-1^ |
| [ATP]_i_ | Concentration of total ATP in IM space | mol (l IM water) ^-1^ |
| [ADP]_i_ | Concentration of total ADP in IM space | mol (l IM water) ^-1^ |
| [AMP]_i_ | Concentration of total AMP in IM space | mol (l IM water) ^-1^ |
| [PI]_i_ | Concentration of inorganic phosphate in IM space | mol (l IM water) ^-1^ |
| [PYR]_i_ | Concentration of pyruvate in IM space | mol (l IM water) ^-1^ |
| [CIT]_i_ | Concentration of citrate in IM space | mol (l IM water) ^-1^ |
| [AKG]_i_ | Concentration of α-ketoglutarate in IM space | mol (l IM water) ^-1^ |
| [SUC]_i_ | Concentration of pyruvate in IM space | mol (l IM water) ^-1^ |
| [FUM]_i_ | Concentration of fumarate in IM space | mol (l IM water) ^-1^ |
| [MAL]_i_ | Concentration of malate in IM space | mol (l IM water) ^-1^ |
| [ASP]_i_ | Concentration of ssparatate in IM space | mol (l IM water) ^-1^ |
| [GLU]_i_ | Concentration of glutamate in IM space | mol (l IM water) ^-1^ |
| Cytoplasm Variables: | | |
| [H^+^]_c_ | Concentration of H^+^ ion in cytoplasm | mol (l cytoplasm water) ^-1^ |
| [K^+^]_c_ | Concentration of K^+^ ion in cytoplasm | mol (l cytoplasm water) ^-1^ |
| [Mg^2+^]_c_ | Concentration of Mg^2+^ ion in cytoplasm | mol (l cytoplasm water) ^-1^ |
| [ATP]_c_ | Concentration of total ATP in cytoplasm | mol (l cytoplasm water) ^-1^ |
| [ADP]_c_ | Concentration of total ADP in cytoplasm | mol (l cytoplasm water) ^-1^ |
| [AMP]_c_ | Concentration of total ADP in cytoplasm | mol (l cytoplasm water) ^-1^ |
| [PI]_c_ | Concentration of inorganic phosphate in cytoplasm | mol (l cytoplasm water) ^-1^ |
| [PYR]_c_ | Concentration of pyruvate in cytoplasm | mol (l cytoplasm water) ^-1^ |
| [CIT]_c_ | Concentration of citrate in cytoplasm | mol (l cytoplasm water) ^-1^ |
| [AKG]_c_ | Concentration of α-ketoglutarate in cytoplasm | mol (l cytoplasm water) ^-1^ |
| [SUC]_c_ | Concentration of pyruvate in cytoplasm | mol (l cytoplasm water) ^-1^ |
| [FUM]_c_ | Concentration of fumarate in cytoplasm | mol (l cytoplasm water) ^-1^ |
| [MAL]_c_ | Concentration of malate in cytoplasm | mol (l cytoplasm water) ^-1^ |
| [ASP]_c_ | Concentration of aspartate in cytoplasm | mol (l cytoplasm water) ^-1^ |
| [GLU]_c_ | Concentration of glutamate in cytoplasm | mol (l cytoplasm water) ^-1^ |
| [CrP]_c_ | Concentration of phosphate creatine in cytoplasm | mol (l cytoplasm water) ^-1^ |
| [Cr]_c_ | Concentration of creatine in cytoplasm | mol (l cytoplasm water) ^-1^ |

Table S2: Reaction and transport fluxes

| **Flux** | **Description** | **Units** |
| --- | --- | --- |
| Mitochondrial Reactions: | |  |
| *J*_C1_ | Complex I | mol s^-1^ (l mito)^-1^ |
| *J*_C3_ | Complex III | mol s^-1^ (l mito)^-1^ |
| *J*_C4_ | Complex IV | mol s^-1^ (l mito)^-1^ |
| *J*_F1_ | F_1_F_0_ ATPase reaction | mol s^-1^ (l mito)^-1^ |
| *J*_ANT_ | Adenine nucleotide translocase | mol s^-1^ (l mito)^-1^ |
| *J*_PIHt_ | Phosphate-hydrogen co-transporter | mol s^-1^ (l mito)^-1^ |
| *J*_Hle_ | Proton leak | mol s^-1^ (l mito)^-1^ |
| *J*_KH_ | Mitochondrial K^+^ / H^+^ exchanger | mol s^-1^ (l mito)^-1^ |
| *J*_pdh_ | Pyruvate dehydrogenase | mol s^-1^ (l mito)^-1^ |
| *J*_cits_ | Citrate synthetase | mol s^-1^ (l mito)^-1^ |
| *J*_acon_ | Aconitase | mol s^-1^ (l mito)^-1^ |
| *J*_isod_ | Isocitrate dehydrogenase | mol s^-1^ (l mito)^-1^ |
| *J*_akgd_ | α-Ketoglutarate dehydrogenase | mol s^-1^ (l mito)^-1^ |
| *J*_scoas_ | Succinyl-CoA synthetase | mol s^-1^ (l mito)^-1^ |
| *J*_sdh_ | Succinate dehydrogenase | mol s^-1^ (l mito)^-1^ |
| *J*_fum_ | Fumarase | mol s^-1^ (l mito)^-1^ |
| *J*_mdh_ | Malate dehydrogenase | mol s^-1^ (l mito)^-1^ |
| *J*_ndk_ | Nucleoside diphosphokinase | mol s^-1^ (l mito)^-1^ |
| *J*_got_ | Glutamate oxaloacetate transaminase (Aspartate Transaminase) | mol s^-1^ (l mito)^-1^ |
| *J*_AKi_ | Mitochondrial adenylate kinase | mol s^-1^ (l mito)^-1^ |
| Mitochondrial Transport Fluxes: | |  |
| *J*_PYRH_ | Pyruvate-H^+^ co-transporter | mol s^-1^ (l mito)^-1^ |
| *J*_GLUH_ | Glutamate-H^+^ co-transporter | mol s^-1^ (l mito)^-1^ |
| *J*_CITMAL_ | Citrate/malate antiporter | mol s^-1^ (l mito)^-1^ |
| *J*_AKGMAL_ | α-Ketoglutarate/malate antiporter | mol s^-1^ (l mito)^-1^ |
| *J*_SUCMAL_ | Succinate/malate antiporter | mol s^-1^ (l mito)^-1^ |
| *J*_MALPI_ | Malate/phosphate antiporter | mol s^-1^ (l mito)^-1^ |
| *J*_ASPGLU_ | Aspartate/glutamate antiporter | mol s^-1^ (l mito)^-1^ |
| *J*_PIt_ | Phosphate transport across outer membrane | mol s^-1^ (l mito)^-1^ |
| *J*_ATPt_ | ATP transport across outer membrane | mol s^-1^ (l mito)^-1^ |
| *J*_ADPt_ | ADP transport across outer membrane | mol s^-1^ (l mito)^-1^ |
| *J*_AMPt_ | AMP transport across outer membrane | mol s^-1^ (l mito)^-1^ |
| *J*_PYRt_ | Pyruvate transport across outer membrane | mol s^-1^ (l mito)^-1^ |
| *J*_CITt_ | Citrate transport across outer membrane | mol s^-1^ (l mito)^-1^ |
| *J*_MALt_ | Malate transport across outer membrane | mol s^-1^ (l mito)^-1^ |
| *J*_AKGt_ | α-Ketoglutarate transport across outer membrane | mol s^-1^ (l mito)^-1^ |
| *J*_SUCt_ | Succinate transport across outer membrane | mol s^-1^ (l mito)^-1^ |
| *J*_GLUt_ | Glutamate transport across outer membrane | mol s^-1^ (l mito)^-1^ |
| *J*_ASPt_ | Aspartate transport across outer membrane | mol s^-1^ (l mito)^-1^ |
| Cytoplasm Reactions: | | |
| *J*_ATPase_ | Cytoplasmic ATP consumption rate | mol s^-1^ (l cyto)^-1^ |
| *J*_AKc_ | Cytoplasmic adenylate kinase | mol s^-1^ (l cyto)^-1^ |
| *J*_CK_ | Creatine kinase | mol s^-1^ (l cyto)^-1^ |

Table S3: Model parameter

| **Parameter** | **Description** | **Value** | **Units** | **Reference** |
| --- | --- | --- | --- | --- |
| Enzyme activity and kinetic constant | | | | |
| *X*_pdh_ | Pyruvate dehydrogenase activity | 1.22 × 10^-1^ | mol s^-1^ (l mito)^-1^ | [4] |
| *X*_cits_ | Citrate synthase activity | 1.16 | mol s^-1^ (l mito)^-1^ | [4] |
| *X*_acon_ | Aconitase activity | 3.21 × 10^-2^ | mol s^-1^ (l mito)^-1^ | [4] |
| *X*_isod_ | Isocitrate dehydrogenase activity | 4.25 × 10^-1^ | mol s^-1^ (l mito)^-1^ | [4] |
| *X*_akgd_ | α-Ketoglutarate dehydrogenase activity | 7.70 × 10^-2^ | mol s^-1^ (l mito)^-1^ | [4] |
| *K_ir_,*_akgd_ | Inhibition constant of NADH in α-Ketoglutarate dehydrogenase reaction | 6.04×10^-7^ | M | [4] |
| *X*_scoas_ | Succinyl-CoA synthetase activity | 5.82 × 10^-1^ | mol s^-1^ (l mito)^-1^ | [4] |
| *X*_sdh_ | Succinate dehydrogenase activity | 6.23 × 10^-2^ | mol s^-1^ (l mito)^-1^ | [4] |
| *X*_fuma_ | Fumarase activity | 7.12 × 10^-3^ | mol s^-1^ (l mito)^-1^ | [4] |
| *X*_mdh_ | Malate dehydrogenase activity | 6.94 × 10^-2^ | mol s^-1^ (l mito)^-1^ | [4] |
| *X*_ndk_ | Nucleoside diphosphokinase activity | 2.65 × 10^-2^ | mol s^-1^ (l mito volume)^-1^ | [4] |
| *X*_got_ | Glutamate oxaloacetate transaminase | 7.96 | mol s^-1^ (l mito)^-1^ | [4] |
| *X*_PYRH_ | PYR^-^/H^+^ co-transporter activity | 4.12 × 10^8^ | mol s^-1^ M^-2^ (l mito)^-1^ | [4] |
| *X*_GLUH_ | GLU^-^/H^+^ co-transporter activity | 3.26 × 10^8^ | mol s^-1^ M^-2^ (l mito volume)^-1^ | [4] |
| *X*_CITMAL_ | HCIT^2-^/MAL^2-^ antiporter activity | 7.31 × 10^1^ | mol s^-1^ M^-2^ (l mito volume)^-1^ | [4] |
| *X*_AKGMAL_ | AKG^2-^/MAL^2-^ antiporter activity | 3.46 × 10^-1^ | mol s^-1^ (l mito volume)^-1^ | [4] |
| *X*_SUCMAL_ | SUC^2-^/MAL^2-^ antiporter activity | 9.54 × 10^1^ | mol s^-1^ M^-2^ (l mito)^-1^ | [4] |
| *X*_MALPI_ | MAL^2-^/PI^2-^ antiporter activity | 1.58 × 10^1^ | mol s^-1^ M^-2^ (l mito)^-1^ | [4] |
| *X*_ASPGLU_ | ASP^-^/HGLU^0^ antiporter activity | 7.48 × 10^-5^ | mol s^-1^ (l mito)^-1^ | [4] |
| *X*_C1_ | Complex I activity | 2.47 × 10^4^ | mol s^-1^ M^-2^ (l mito)^-1^ | [4] |
| *X*_C3_ | Complex III activity | 6.65 × 10^-1^ | mol s^-1^ M^-3/2^ (l mito)^-1^ | [4] |
| *k*_PI,1_ | Complex III/PI parameter | 2.81 × 10^-5^ | M | [4] |
| *k*_PI,2_ | Complex III/PI parameter | 3.14 × 10^-3^ | M | [4] |
| *X*_C4_ | Complex IV activity | 9.93 × 10^-5^ | mol s^-1^ M^-1^ (l mito)^-1^ | [4] |
| *X*_F1_ | F_o_F_1_-ATPase activity | 5.95 × 10^3^ | mol s^-1^ M^-1^ (l mito)^-1^ | [4] |
| *X*_ANT_ | ANT activity | 1.52 × 10^-1^ | unitless | [1] |
| *X*_PIHt_ | H_2_PO_4_^-^/H^+^ co-transporter activity | 2.01 × 10^7^ | mol s^-1^ M^-1^ (l mito)^-1^ | [4] |
| *k*_PIHt_ | H_2_PO_4_^-^/H^+^ co-transporter parameter | 1.01 × 10^-3^ | M | [4] |
| *X*_KH_ | K^+^/H^+^ antiporter activity | 5.65 × 10^6^ | mol s^-1^ M^-2^ (l mito)^-1^ | [4] |
| *X*_Hle_ | Proton leak activity | 3.05 × 10^2^ | mol s^-1^ mV^-1^ M^-1^ (l mito)^-1^ | [4] |
| *X*_AKi_ | IM space adenylate kinase activity | 1 × 10^10^ | mol s^-1^ M^-2^ (l mito)^-1^ | - |
| *X*_AKc_ | Cytoplasmic adenylate kinase activity | 1 × 10^10^ | mol s^-1^ M^-2^ (l cyto)^-1^ | - |
| *X*_CKc_ | Cytoplasmic creatine kinase | 1 × 10^10^ | mol s^-1^ M^-2^ (l cyto)^-1^ | - |
| Oxygen Transport Parameters | | | | |
| α_1_ | Plasma O_2_ solubility | 1.30 × 10^-6^ | M mm Hg^-1^ | [5] |
| α_2_ | Interstitial fluid O_2_ solubility | 1.25 × 10^-6^ | M mm Hg^-1^ | [6] |
| α_3_ | Myocyte O_2_ solubility | 1.74 × 10^-6^ | M mm Hg^-1^ | [7] |
| *PS*_12_ | Capillary wall PS product | 50 | ml s^-1^ (ml tissue)^-1^ | [8, 9] |
| *PS*_23_ | Myocyte fiber PS product | 10 | ml s^-1^ (ml tissue)^-1^ | [10] |
| *Hct* | Hematocrit | 0.45 | unitless | [2] |
| *C*_Hb_ | Oxyhemoglobin binding site concentration | 0.0213 | mol (l RBC)^-1^ | [5] |
| *P*_50,Hb_ | Hemoglobin half-saturation *P*_O2_ | 30.0 | mm Hg | [11] |
| *n_H_* | Hemoglobin Hill coefficient | 2.55 | unitless | [11] |
| *C*_Mb_ | Myoglobin saturation | 200 × 10^-6^ | mol (l cell)^-1^ | [2] |
| *P*_50,Mb_ | Myoglobin half-saturation *P*_O2_ | 2.39 | mm Hg | [12] |
| *P*_input_ | Arterial oxygen tension | 100 | mm Hg | [2] |
| Physicochemical Parameters: | | | | |
| *RT* | Gas constant times temperature | 2.5775 | kJ mol^-1^ | –^a^ |
| *F* | Faraday’s constant | 0.096484 | kJ mol^-1^ mV^-1^ | –^a^ |
| Structure/Volume Parameters: | | | | |
| ρ | Tissue density | 1.053 | g (ml tissue)^-1^ | [13] |
| *L* | Capillary length | 550 | μm | [14] |
| *V*_1_ | Capillary blood volume | 0.05 | ml (ml tissue)^-1^ | [15, 16] |
| *V*_2_ | Interstitial volume | 0.17585 | ml (ml tissue)^-1^ | [13] |
| *V*_3_ | Myocyte volume | 0.73078 | ml (ml tissue)^-1^ | [13] |
| *V*_cyto_ | Cytoplasm Volume | 0.894 | (l cytoplasm) (l cell) ^-1^ | [17] |
| *V*_mito_ | Mitochondrial Volume | 0.056 | (l mito) (l cell) ^-1^ | [18] |
| *W*_x_ | Matrix water space fraction | 0.6514 | (l water) (l mito) ^-1^ | [13, 19] |
| *W*_i_ | IM space water fraction | 0.0724 | (l water) (l mito) ^-1^ | [13, 19] |
| *W_c_* | Cytoplasm water fraction | 0.8425 | (l water) (l cyto) ^-1^ | [13] |
| γ | Outer membrane area per mito volume | 5.99 | μm^-1^ | [20] |
| *ρ_m_* | Protein density of mitochondria | 2.725 × 10^5^ | (mg Protein) (l mito)^-1^ | [13] |
| Mitochondrial Model Parameters: | | | | |
| *n*_A_ | H^+^ stoich. coef. for F_1_F_0_-ATPase | 3 | unitless | [21] |
| *p*_PI_ | Mitochondrial membrane permeability to inorganic phosphate | 327 | μm sec^-1^ | [22] |
| *p*_A_ | Mitochondrial outer membrane permeability to nucleotides | 85.0 | μm sec^-1^ | [23] |
| *k*_O2_ | Kinetic constant for complex IV | 1.2 × 10^-4^ | M | [22]^d^ |
| *C*_IM_ | Capacitance of inner membrane | 6.75 × 10^-6^ | mol (l mito) ^-1^ mV^-1^ | [19, 24] |
| *B_x_* | Matrix buffering parameter | 0.02 | M | [25, 26]^b^ |
| *K_Bx_* | Matrix buffering parameter | 1 × 10^-7^ | M | [25, 26]^b^ |
| Fixed Concentrations and Concentration Pools: | | | | |
| NAD_tot_ | Total matrix NAD(H) concentration | 2.97 | mol (l matrix water) ^-1^ | [22]^c^ |
| Q_tot_ | Total matrix ubiquinol concentration | 1.35 | mol (l matrix water) ^-1^ | [22]^c^ |
| cytC_tot_ | Total IM cytochrome c concentration | 2.70 | mol (l IM water) ^-1^ | [22]^c^ |
| A_tot_ | Total matrix ATP+ADP concentration | 10 | mol (l matrix water) ^-1^ | [22]^c^ |
| CR_tot_ | Total Cr+CrP concentration | 42.7 | mol (l cytoplasm water) ^-1^ | [27] |
| [CO_2_tot]_x_ | Total CO_2_ concentration in the matrix | 21.4 × 10^-3^ | Molar | [28] |
| Standard Gibbs Free Energy or Equilibrium Constants of Reference Reactions:^d^ | | | | |
| $\Delta_{r}G_{\text{C1}}^{0}$ | Complex I | -109.7 | kJ mol^-1^ | [4] |
| $\Delta_{r}G_{\text{C3}}^{0}$ | Complex III | 46.69 | kJ mol^-1^ | [4] |
| $\Delta_{r}G_{\text{C4}}^{0}$ | Complex IV | -202.2 | kJ mol^-1^ | [4] |
| $\Delta_{r}G_{\text{F1}}^{0}$ | F_o_F_1_-ATPase | -4.51 | kJ mol^-1^ | [4] |
| $\Delta_{r}G_{\text{pdh}}^{0}$ | Pyruvate dehydrogenase | 19.59 | kJ mol^-1^ | [4] |
| $\Delta_{r}G_{\text{cits}}^{0}$ | Citrate synthase | 42.36 | kJ mol^-1^ | [4] |
| $\Delta_{r}G_{\text{acon}}^{0}$ | Aconitase | 12.82 | kJ mol^-1^ | [4] |
| $\Delta_{r}G_{\text{isod}}^{0}$ | Isocitrate dehydrogenase | 91.75 | kJ mol^-1^ | [4] |
| $\Delta_{r}G_{\text{akgd}}^{0}$ | α-Ketoglutarate dehydrogenase | 12.82 | kJ mol^-1^ | [4] |
| $\Delta_{r}G_{\text{scoas}}^{0}$ | Succinyl-CoA synthetase | 47.61 | kJ mol^-1^ | [4] |
| $\Delta_{r}G_{\text{sdh}}^{0}$ | Succinate dehydrogenase | -1.35 | kJ mol^-1^ | [4] |
| $\Delta_{r}G_{\text{fum}}^{0}$ | Fumarase | -3.60 | kJ mol^-1^ | [4] |
| $\Delta_{r}G_{\text{mdh}}^{0}$ | Malate dehydrogenase | 69.12 | kJ mol^-1^ | [4] |
| $\Delta_{r}G_{\text{ndk}}^{0}$ | Nucleoside diphosphokinase | 0 | kJ mol^-1^ | [4] |
| $\Delta_{r}G_{\text{got}}^{0}$ | Glutamate Oxaloacetate Transaminase | -1.47 | kJ mol^-1^ | [4] |
| $K_{eq,\text{AK}}^{o}$ | Adenylate kinase | 3.97 × 10^-1^ | unitless | [1] |
| $K_{eq,\text{CK}}^{o}$ | Creatine kinase | 3.57 × 10^8^ | M^-1^ | [1] |

^a^Standard physicochemical constants

^b^Values are adjusted to match experimental data.

^c^Value used is taken from previous modeling studies, not direct experimental measure.

^d^Values used are calculated for reference reactions at physiological temperature (310.15 K) and ionic strength (0.17 M) based on standard thermodynamic data collected from the Alberty’s book [29] and NIST database [30]. Please refer to Appendices of [4] and [1] for detailed computation procedures.

## S2. Computational model

The model is mathematically described by using the differential equations listed below. The oxidative phosphorylation component of the model is derived from previously published work [19, 31, 32]. The details behind the TCA cycle enzyme kinetic schemes are provided in Appendix C of our previously published work [4]. Here the subscripts “x”, “i”, and “c” on variable names denote matrix, intermembrane, and cytoplasmic (extra-mitochondrial) spaces, respectively. For example [ATP]_x_ denotes matrix ATP concentration while [ATP]_c_ denotes ATP concentration in the cytoplasm or buffer space for an isolated mitochondria experiment.

### S2.1. Differential equations

The differential equations are grouped into equations for oxygen concentration, membrane potential, mitochondrial matrix variables, intermembrane space variables, and cytoplasm variables. The time derivatives of free [H^+^], [Mg^2+^], and [K^+^] are treated separately.

**Oxygen concentration:**

$$\partial C_{\text{O}_{2}\text{,capillary}}/\partial t=-\frac{\rho GL}{V_{1}}\partial C_{\text{O}_{2}\text{,capillary}}/\partial x-\frac{\alpha_{1}PS_{12}}{V_{1}}(P_{\text{O}_{2}\text{,capillary}}-P_{\text{O}_{2}\text{,interstitium}})$$

$$\partial C_{\text{O}_{2}\text{,interstitium}}/\partial t=-\frac{\alpha_{1}PS_{12}}{V_{2}}(P_{\text{O}_{2}\text{,capillary}}-P_{\text{O}_{2}\text{,interstitium}})-\frac{\alpha_{1}PS_{23}}{V_{2}}(P_{\text{O}_{2}\text{,interstitium}}-P_{\text{O}_{2}\text{,cell}})$$

$$\partial C_{\text{O}_{2}\text{,cell}}/\partial t=-\frac{\alpha_{1}PS_{23}}{V_{2}}(P_{\text{O}_{2}\text{,capillary}}-P_{\text{O}_{2}\text{,cell}})-V_{\text{mito}}\left(\frac{J_{C4}}{2}\right)$$

where

$$C_{\text{O}_{2}\text{,capillary}}=\alpha_{1}P_{\text{O}_{2}\text{,capillary}}+HctC_{\text{Hb}}S_{\text{Hb}}$$

$$S_{\text{Hb}}=\frac{P_{\text{O}_{2}\text{,capillary}}^{n_{H}}}{P_{\text{O}_{2}\text{,capillary}}^{n_{H}}+P_{\text{50,Hb}}^{n_{H}}}$$

$$C_{\text{O}_{2}\text{,cell}}=\alpha_{3}P_{\text{O}_{2}\text{,cell}}+C_{\text{Mb}}S_{\text{Mb}}$$

$S_{\text{Mb}}=\frac{P_{\text{O}_{2}\text{,cell}}}{P_{\text{O}_{2}\text{,cell}}+P_{\text{50,Mb}}}$.

**Mitochondrial Inner Membrane Electrical Potential:**

$$\partial\Delta\Psi/\partial t=(+4J_{\text{C}1}+2J_{\text{C3}}+4J_{\text{C4}}-n_{\text{A}}J_{\text{F1}}-J_{\text{ANT}}-J_{\text{Hle}}+J_{\text{ASPGLU}})/C_{\text{ΙΜ}}$$

**Mitochondrial Matrix:**

$$\partial[\text{ATP}]_{\text{x}}/\partial t=(+J_{\text{ndk}}+J_{\text{F1}}-J_{\text{ANT}})/W_{\text{x}}$$

$$\partial[\text{ADP}]_{\text{x}}/\partial t=(-J_{\text{ndk}}-J_{\text{F1}}+J_{\text{ANT}})/W_{\text{x}}$$

$$\partial[\text{AMP}]_{\text{x}}/\partial t=0/W_{\text{x}}$$

$$\partial[\text{GTP}]_{\text{x}}/\partial t=(+J_{\text{scoas}}-J_{\text{ndk}})/W_{\text{x}}$$

$$d[\text{GDP}]_{\text{x}}/dt=(-J_{\text{scoas}}+J_{\text{ndk}})/W_{\text{x}}$$

$$\partial[\text{PI}]_{\text{x}}/\partial t=(-J_{\text{scoas}}-J_{\text{F1}}+J_{\text{PIHt}}-J_{\text{SUCPI}}-J_{\text{MALPI}})/W_{\text{x}}$$

$$\partial[\text{NADH}]_{\text{x}}/\partial t=(+J_{\text{pdh}}+J_{\text{isod}}+J_{\text{akgd}}+J_{\text{mdh}}-J_{\text{C1}})/W_{\text{x}}$$

$$\partial[\text{QH}_{2}]_{\text{x}}/\partial t=(+J_{\text{sdh}}+J_{\text{C1}}-J_{\text{C3}})/W_{\text{x}}$$

$$\partial[\text{PYR}]_{\text{x}}/\partial t=(-J_{\text{pdh}}+J_{\text{PYRH}})/W_{\text{x}}$$

$$\partial[\text{ACCOA}]_{\text{x}}/\partial t=(-J_{\text{cits}}+J_{\text{pdh}})/W_{\text{x}}$$

$$\partial[\text{CIT}]_{\text{x}}/\partial t=(+J_{\text{cits}}-J_{\text{acon}}+J_{\text{CITMAL}})/W_{\text{x}}$$

$$\partial[\text{ICIT}]_{\text{x}}/\partial t=(+J_{\text{acon}}-J_{\text{isod}})/W_{\text{x}}$$

$$\partial[\text{AKG}]_{\text{x}}/\partial t=(+J_{\text{isod}}-J_{\text{akgd}}-J_{\text{got}}+J_{\text{AKGMAL}})/W_{\text{x}}$$

$$\partial[\text{SCOA}]_{\text{x}}/\partial t=(+J_{\text{akgd}}-J_{\text{scoas}})/W_{\text{x}}$$

$$\partial[\text{COASH}]_{\text{x}}/\partial t=(-J_{\text{pdh}}-J_{\text{akgd}}+J_{\text{scoas}}+J_{\text{cits}})/W_{\text{x}}$$

$$\partial[\text{SUC}]_{\text{x}}/\partial t=(+J_{\text{scoas}}-J_{\text{sdh}}+J_{\text{SUCPI}}-J_{\text{FUMSUC}})/W_{\text{x}}$$

$$\partial[\text{FUM}]_{\text{x}}/\partial t=(+J_{\text{sdh}}-J_{\text{fum}}+J_{\text{FUMSUC}})/W_{\text{x}}$$

$$\partial[\text{MAL}]_{\text{x}}/\partial t=(+J_{\text{fum}}-J_{\text{mdh}}+J_{\text{MALPI}}-J_{\text{AKGMAL}}-J_{\text{CITMAL}})/W_{\text{x}}$$

$$\partial[\text{OAA}]_{\text{x}}/\partial t=(-J_{\text{cits}}+J_{\text{mdh}}+J_{\text{got}})/W_{\text{x}}$$

$$\partial[\text{GLU}]_{\text{x}}/\partial t=(+J_{\text{got}}+J_{\text{GLUH}}-J_{\text{ASPGLU}})/W_{\text{x}}$$

$$\partial[\text{ASP}]_{\text{x}}/\partial t=(-J_{\text{got}}+J_{\text{ASPGLU}})/W_{\text{x}}$$

$\partial[\text{CO}_{2}\text{tot}]_{\text{x}}/\partial t=0/W_{\text{x}}$.

**Mitochondrial Inter-Membrane Space:**

$$\partial[\text{Cred}]_{\text{i}}/\partial t=(+2J_{\text{C3}}-2J_{\text{C4}})/W_{\text{i}}$$

$$\partial[\text{ATP}]_{\text{i}}/\partial t=(+J_{\text{ATP}}+J_{\text{ANT}}+J_{\text{AKi}})/W_{\text{i}}$$

$$\partial[\text{ADP}]_{\text{i}}/\partial t=(+J_{\text{ADP}}-J_{\text{ANT}}-2J_{\text{AKi}})/W_{\text{i}}$$

$$\partial[\text{AMP}]_{\text{i}}/\partial t=(J_{\text{AMPt}}+J_{\text{AKi}})/W_{\text{i}}$$

$$\partial[\text{PI}]_{\text{i}}/\partial t=(-J_{\text{PIHt}}+J_{\text{PIt}}+J_{\text{MALPI}}+J_{\text{SUCPI}})/W_{\text{i}}$$

$$\partial[\text{PYR}]_{\text{i}}/\partial t=(-J_{\text{PYRH}}+J_{\text{PYRt}})/W_{\text{i}}$$

$$\partial[\text{CIT}]_{\text{i}}/\partial t=(-J_{\text{CITMAL}}+J_{\text{CITt}})/W_{\text{i}}$$

$$\partial[\text{ICIT}]_{\text{i}}/\partial t=(-J_{\text{ICITMAL}}+J_{\text{ICITt}})/W_{\text{i}}$$

$$\partial[\text{AKG}]_{\text{i}}/\partial t=(-J_{\text{AKGMAL}}+J_{\text{AKGt}})/W_{\text{i}}$$

$$\partial[\text{SUC}]_{\text{i}}/\partial t=(-J_{\text{SUCPI}}+J_{\text{SUCt}}+J_{\text{FUMSUC}})/W_{\text{i}}$$

$$\partial[\text{FUM}]_{\text{i}}/\partial t=(-J_{\text{FUMPI}}+J_{\text{FUMt}})/W_{\text{i}}$$

$$\partial[\text{MAL}]_{\text{i}}/\partial t=(-J_{\text{MALPI}}+J_{\text{MALt}}+J_{\text{AKGMAL}}+J_{\text{CITMAL}})/W_{\text{i}}$$

$$\partial[\text{GLU}]_{\text{i}}/\partial t=(-J_{\text{GLUH}}+J_{\text{ASPGLU}}+J_{\text{GLUt}})/W_{\text{i}}$$

$\partial[\text{ASP}]_{\text{i}}/\partial t=(-J_{\text{ASPGLU}}+J_{\text{ASPt}})/W_{\text{i}}$.

Cytoplasm:

$$\partial[\text{ATP}]_{\text{c}}/\partial t=(-(V_{\text{cyto}}/V_{\text{mito}})J_{\text{ATPt}}-J_{\text{ATPase}}+J_{\text{CKc}}+J_{\text{AKc}})/W_{\text{c}}$$

$$\partial[\text{ADP}]_{\text{c}}/\partial t=(-(V_{\text{cyto}}/V_{\text{mito}})J_{\text{ADPt}}+J_{\text{ATPase}}-J_{\text{CKc}}-2J_{\text{AKc}})/W_{\text{c}}$$

$$\partial[\text{AMP}]_{\text{c}}/\partial t=(-(V_{\text{cyto}}/V_{\text{mito}})J_{\text{AMPt}}+J_{\text{AKc}})/W_{\text{c}}$$

$$\partial[\text{PI}]_{\text{c}}/\partial t=(-(V_{\text{cyto}}/V_{\text{mito}})J_{\text{PIt}}+J_{\text{AKc}})/W_{\text{c}}$$

$$\partial[\text{PYR}]_{\text{c}}/\partial t=-(V_{\text{cyto}}/V_{\text{mito}})J_{\text{PYRt}}/W_{\text{c}}$$

$$\partial[\text{CIT}]_{\text{c}}/\partial t=-(V_{\text{cyto}}/V_{\text{mito}})J_{\text{CITt}}/W_{\text{c}}$$

$$\partial[\text{AKG}]_{\text{c}}/\partial t=-(V_{\text{cyto}}/V_{\text{mito}})J_{\text{AKGt}}/W_{\text{c}}$$

$$\partial[\text{SUC}]_{\text{c}}/\partial t=-(V_{\text{cyto}}/V_{\text{mito}})J_{\text{SUCt}}/W_{\text{c}}$$

$$\partial[\text{FUM}]_{\text{c}}/\partial t=-(V_{\text{cyto}}/V_{\text{mito}})J_{\text{FUMt}}/W_{\text{c}}$$

$$\partial[\text{MAL}]_{\text{c}}/\partial t=-(V_{\text{cyto}}/V_{\text{mito}})J_{\text{MALt}}/W_{\text{c}}$$

$$\partial[\text{GLU}]_{\text{c}}/\partial t=-(V_{\text{cyto}}/V_{\text{mito}})J_{\text{GLUt}}/W_{\text{c}}$$

$$\partial[\text{ASP}]_{\text{c}}/\partial t=-(V_{\text{cyto}}/V_{\text{mito}})J_{\text{ASPt}}/W_{\text{c}}$$

$\partial[\text{PCr}]_{\text{c}}/\partial t=-J_{\text{CKc}}/W_{\text{c}}$.

Assuming constant total concentrations NAD_tot_, Q_tot_, cytC_tot_, and A_tot_ for nicotinamide nucleotides, ubiquinol, and cytochrome c, we compute concentrations of the following reactants as:

$$[\text{NAD]}_{\text{x}}=\text{ NAD}_{\text{tot}}-[\text{NADH]}_{\text{x}}$$

$$[\text{COQ]}_{\text{x}}=\text{ Q}_{\text{tot}}-[\text{QH}_{2}\text{]}_{\text{x}}$$

$[\text{Cox]}_{\text{i}}=\text{ cytC}_{\text{tot}}-[\text{Cred]}_{\text{i}}$.

Concentrations of cytoplasmic H^+^, Mg^2+^, and K^+^ are assumed to be fixed at buffer conditions or physiological in vivo values. Since the outer membrane is highly permeable to hydrogen ions and cations, we assume here$\text{[H}^{\text{+}}\text{]}_{\text{i}}\text{ = [H}^{\text{+}}\text{]}_{\text{c}}$, $\text{[Mg}^{\text{2+}}\text{]}_{\text{i}}\text{ = [Mg}^{\text{2+}}\text{]}_{\text{c}}$, and$\text{[K}^{\text{+}}\text{]}_{\text{i}}\text{ = [K}^{\text{+}}\text{]}_{\text{c}}$.

The rate of change of free [H^+^], [Mg^2+^] and [K^+^] in the mitochondrial matrix can be calculated based on mass conservation. Mathematical expressions for time derivatives of [H^+^], [Mg^2+^], and [K^+^] are listed below. For detailed description and derivation of these expressions, please refer to Chapter 6 of Beard and Qian [33].

$$\begin{matrix} \frac{\partial[\text{H}^{\text{+}}]}{\partial t}\text{ = }\left[\left(\frac{\partial[\text{K}_{bound}]}{\partial[\text{Mg}^{\text{2+}}]}\cdot\frac{\partial[\text{Mg}_{bound}]}{\partial[\text{K}^{\text{+}}]}-\alpha_{Mg}\alpha_{K}\right)\Phi^{H}\right. \\ \begin{matrix} & & \end{matrix}\text{+ }\left(\alpha_{K}\frac{\partial[\text{H}_{bound}]}{\partial[\text{Mg}^{\text{2+}}]}-\frac{\partial[\text{H}_{bound}]}{\partial[\text{K}^{\text{+}}]}\cdot\frac{\partial[\text{K}_{bound}]}{\partial[\text{Mg}^{\text{2+}}]}\right)\Phi^{Mg} \\ \begin{matrix} & & \end{matrix}\text{+ }\left.\left(\alpha_{Mg}\frac{\partial[\text{H}_{bound}]}{\partial[\text{K}^{\text{+}}]}-\frac{\partial[\text{H}_{bound}]}{\partial[\text{Mg}^{\text{2+}}]}\cdot\frac{\partial[\text{Mg}_{bound}]}{\partial[\text{K}^{\text{+}}]}\right)\Phi^{K}\right]/D \end{matrix}$$

$$\begin{matrix} \frac{\partial[\text{Mg}^{\text{2+}}]}{\partial t}\text{ = }\left[\left(\alpha_{K}\frac{\partial[\text{Mg}_{bound}]}{\partial[\text{H}^{\text{+}}]}-\frac{\partial[\text{K}_{bound}]}{\partial[\text{H}^{\text{+}}]}\cdot\frac{\partial[\text{Mg}_{bound}]}{\partial[\text{K}^{\text{+}}]}\right)\Phi^{H}\right. \\ \begin{matrix} & & \end{matrix}\text{ + }\left(\frac{\partial[\text{K}_{bound}]}{\partial[\text{H}^{\text{+}}]}\cdot\frac{\partial[\text{H}_{bound}]}{\partial[\text{K}^{\text{+}}]}-\alpha_{H}\alpha_{K}\right)\Phi^{Mg} \\ \begin{matrix} & & \end{matrix}\text{ + }\left.\left(\alpha_{H}\frac{\partial[\text{Mg}_{bound}]}{\partial[\text{K}^{\text{+}}]}-\frac{\partial[\text{H}_{bound}]}{\partial[\text{K}^{\text{+}}]}\cdot\frac{\partial[\text{Mg}_{bound}]}{\partial[\text{H}^{\text{+}}]}\right)\Phi^{K}\right]/D \end{matrix}$$

$\begin{matrix} \frac{\partial[\text{K}^{\text{+}}]}{\partial t}\text{ = }\left[\left(\alpha_{Mg}\frac{\partial[\text{K}_{bound}]}{\partial[\text{H}^{\text{+}}]}-\frac{\partial[\text{K}_{bound}]}{\partial[\text{Mg}^{\text{2+}}]}\cdot\frac{\partial[\text{Mg}_{bound}]}{\partial[\text{H}^{\text{+}}]}\right)\Phi^{H}\right. \\ \begin{matrix} & & \end{matrix}\text{+ }\left(\alpha_{H}\frac{\partial[\text{K}_{bound}]}{\partial[\text{Mg}^{\text{2+}}]}-\frac{\partial[\text{K}_{bound}]}{\partial[\text{H}^{\text{+}}]}\cdot\frac{\partial[\text{H}_{bound}]}{\partial[\text{Mg}^{\text{2+}}]}\right)\Phi^{Mg} \\ \begin{matrix} & & \end{matrix}\text{+ }\left.\left(\frac{\partial[\text{Mg}_{bound}]}{\partial[\text{H}^{\text{+}}]}\cdot\frac{\partial[\text{H}_{bound}]}{\partial[\text{Mg}^{\text{2+}}]}-\alpha_{H}\alpha_{Mg}\right)\Phi^{K}\right]/D \end{matrix}$.

The binding polynomials are calculated as

$P_{i}\left([\text{H}^{+}],[\text{Mg}^{2+}],[\text{K}^{+}]\right)\text{ = }1+[\text{H}^{+}]/K_{i}^{H}+[\text{Mg}^{2+}]/K_{i}^{Mg}+[\text{K}^{+}]/K_{i}^{K}$.

The partial derivatives of total concentrations of bound [H^+^], [Mg^2+^], and [K^+^] are expressed as:

$$\frac{\partial[\text{H}_{bound}]}{\partial[\text{Mg}^{2+}]}\text{ = }-\sum_{i=1}^{N_{r}} \frac{[\text{L}_{i}][\text{H}^{\text{+}}]/K_{i}^{H}}{K_{i}^{Mg}\left(P_{i}([\text{H}^{\text{+}}],[\text{Mg}^{\text{2+}}],[\text{K}^{\text{+}}])\right)^{2}}$$

$$\frac{\partial[\text{H}_{bound}]}{\partial[\text{K}^{+}]}\text{ = }-\sum_{i=1}^{N_{r}} \frac{[\text{L}_{i}][\text{H}^{\text{+}}]/K_{i}^{H}}{K_{i}^{K}\left(P_{i}([\text{H}^{\text{+}}],[\text{Mg}^{\text{2+}}],[\text{K}^{\text{+}}])\right)^{2}}$$

$$\frac{\partial[\text{H}_{bound}]}{\partial[\text{H}^{+}]}\text{ = }\sum_{i=1}^{N_{r}} \frac{[\text{L}_{i}]\left(1+[\text{Mg}^{\text{2+}}]/K_{i}^{Mg}+[\text{K}^{\text{+}}]/K_{i}^{K}\right)}{K_{i}^{H}\left(P_{i}([\text{H}^{\text{+}}],[\text{Mg}^{\text{2+}}],[\text{K}^{\text{+}}])\right)^{2}}$$

$$\frac{\partial[\text{Mg}_{bound}]}{\partial[\text{H}^{+}]}\text{ = }-\sum_{i=1}^{N_{r}} \frac{[\text{L}_{i}][\text{Mg}^{\text{2+}}]/K_{i}^{Mg}}{K_{i}^{H}\left(P_{i}([\text{H}^{\text{+}}],[\text{Mg}^{\text{2+}}],[\text{K}^{\text{+}}])\right)^{2}}$$

$$\frac{\partial[\text{Mg}_{bound}]}{\partial[\text{K}^{+}]}\text{ = }-\sum_{i=1}^{N_{r}} \frac{[\text{L}_{i}][\text{Mg}^{\text{2+}}]/K_{i}^{Mg}}{K_{i}^{K}\left(P_{i}([\text{H}^{\text{+}}],[\text{Mg}^{\text{2+}}],[\text{K}^{\text{+}}])\right)^{2}}$$

$$\frac{\partial[\text{Mg}_{bound}]}{\partial[\text{Mg}^{2+}]}\text{ = }\sum_{i=1}^{N_{r}} \frac{[\text{L}_{i}]\left(1+[\text{H}^{\text{+}}]/K_{i}^{H}+[\text{K}^{\text{+}}]/K_{i}^{K}\right)}{K_{i}^{Mg}\left(P_{i}([\text{H}^{\text{+}}],[\text{Mg}^{\text{2+}}],[\text{K}^{\text{+}}])\right)^{2}}$$

$$\frac{\partial[\text{K}_{bound}]}{\partial[\text{H}^{+}]}\text{ = }-\sum_{i=1}^{N_{r}} \frac{[\text{L}_{i}][\text{K}^{\text{+}}]/K_{i}^{K}}{K_{i}^{H}\left(P_{i}([\text{H}^{\text{+}}],[\text{Mg}^{\text{2+}}],[\text{K}^{\text{+}}])\right)^{2}}$$

$$\frac{\partial[\text{K}_{bound}]}{\partial[\text{Mg}^{2+}]}\text{ = }-\sum_{i=1}^{N_{r}} \frac{[\text{L}_{i}][\text{K}^{\text{+}}]/K_{i}^{K}}{K_{i}^{Mg}\left(P_{i}([\text{H}^{\text{+}}],[\text{Mg}^{\text{2+}}],[\text{K}^{\text{+}}])\right)^{2}}$$

$$\frac{\partial[\text{K}_{bound}]}{\partial[\text{K}^{+}]}\text{ = }\sum_{i=1}^{N_{r}} \frac{[\text{L}_{i}]\left(1+[\text{H}^{\text{+}}]/K_{i}^{H}+[\text{Mg}^{\text{2+}}]/K_{i}^{Mg}\right)}{K_{i}^{K}\left(P_{i}([\text{H}^{\text{+}}],[\text{Mg}^{\text{2+}}],[\text{K}^{\text{+}}])\right)^{2}}.$$

The flux terms for H^+^, Mg^2+^, and K^+^ are:

$$\Phi^{H}\text{ = }-\sum_{i=1}^{N_{r}} \frac{\partial[\text{H}_{bound}]}{\partial[\text{L}_{i}]}\frac{d[\text{L}_{i}]}{dt}\text{ + }\sum_{k=1}^{N_{f}} n_{k}J_{k}\text{ + }J_{t}^{H}$$

$$\Phi^{Mg}\text{ = }-\sum_{i=1}^{N_{r}} \frac{\partial[\text{Mg}_{bound}]}{\partial[\text{L}_{i}]}\frac{d[\text{L}_{i}]}{dt}\text{ + }J_{t}^{Mg}$$

$$\Phi^{K}\text{ = }-\sum_{i=1}^{N_{r}} \frac{\partial[\text{K}_{bound}]}{\partial[\text{L}_{i}]}\frac{d[\text{L}_{i}]}{dt}\text{ + }J_{t}^{K}.$$

where *N_r_* is the number of reactants, *N_f_* is the number of reactions, *n_k_* is the stoichiometric coefficient of *k^th^* reaction, *J_k_* is the flux of *k^th^* reaction, *J_t_^H^* (*J_t_^Mg^*, *J_t_^K^*) is the transport flux of [H^+^] ([Mg^2+^], [K^+^]) into the system. In the current model, for the mitochondrial matrix, we have

$$\sum_{k=1}^{N_{f}} n_{k}J_{k}\text{ = }\left(-J_{\text{pdh}}+2J_{\text{cits}}-J_{\text{akgd}}+J_{\text{scoas}}+J_{\text{mdh}}\right)/W_{\text{x}}$$

$$\begin{matrix} J_{t}^{H}\text{ = }\left(J_{\text{PYRH}}\right.+J_{\text{GLUH}}+J_{\text{CITMAL}}-J_{\text{ASPGLU}}-5J_{\text{C1}}-2J_{\text{C3}}-4J_{\text{C4}}+(n_{A}-1)J_{\text{F1}} \\ \begin{matrix} & \end{matrix}\text{ }+2J_{\text{PIHt}}+J_{\text{Hle}}-\left.J_{\text{KH}}\right)/W_{\text{x}} \end{matrix}$$

$$J_{t}^{Mg}\text{ = }0$$

$J_{t}^{K}\text{ = }J_{\text{KH}}/W_{\text{x}}$.

The buffering terms are:

$$\alpha_{H}\text{ = }1\text{ + }\frac{\partial[\text{H}_{bound}]}{\partial[\text{H}^{\text{+}}]}\text{ + }\frac{B_{x}}{K_{Bx}\left(1+[\text{H}^{\text{+}}]/K_{Bx}\right)^{2}}$$

$$\alpha_{Mg}\text{ = }1\text{ + }\frac{\partial[\text{Mg}_{bound}]}{\partial[\text{Mg}^{\text{2+}}]}$$

$$\alpha_{K}\text{ = }1\text{ + }\frac{\partial[\text{K}_{bound}]}{\partial[\text{K}^{\text{+}}]}$$

The denominator term in the time derivatives of [H^+^], [Mg^2+^], and [K^+^] is:

$\begin{matrix} D\text{ = }\alpha_{H}\frac{\partial[\text{K}_{bound}]}{\partial[\text{Mg}^{\text{2+}}]}\cdot\frac{\partial[\text{Mg}_{bound}]}{\partial[\text{K}^{\text{+}}]}\text{ + }\alpha_{K}\frac{\partial[\text{H}_{bound}]}{\partial[\text{Mg}^{\text{2+}}]}\cdot\frac{\partial[\text{Mg}_{bound}]}{\partial[\text{H}^{\text{+}}]} \\ \begin{matrix} & \end{matrix}\text{ + }\alpha_{Mg}\frac{\partial[\text{H}_{bound}]}{\partial[\text{K}^{\text{+}}]}\cdot\frac{\partial[\text{K}_{bound}]}{\partial[\text{H}^{\text{+}}]}\text{ }-\text{ }\alpha_{Mg}\alpha_{K}\alpha_{H} \\ \begin{matrix} & \end{matrix}\text{ }-\frac{\partial[\text{H}_{bound}]}{\partial[\text{K}^{\text{+}}]}\cdot\frac{\partial[\text{K}_{bound}]}{\partial[\text{Mg}^{\text{2+}}]}\cdot\frac{\partial[\text{Mg}_{bound}]}{\partial[\text{H}^{\text{+}}]} \\ \begin{matrix} & \end{matrix}\text{ }-\frac{\partial[\text{H}_{bound}]}{\partial[\text{Mg}^{\text{2+}}]}\cdot\frac{\partial[\text{Mg}_{bound}]}{\partial[\text{K}^{\text{+}}]}\cdot\frac{\partial[\text{K}_{bound}]}{\partial[\text{H}^{\text{+}}]}. \end{matrix}$.

### S2.2. Flux expressions

**Mathematical expressions for oxidative phosphorylation fluxes**

Complex I flux:

$J_{\text{C1}}=X_{\text{C1}}\left(K_{eq,\text{C1}}[\text{NADH}]_{\text{x}}[\text{COQ}]_{\text{x}}-[\text{NAD}]_{\text{x}}[\text{QH}_{2}]_{\text{x}}\right)$,

where $K_{eq,\text{C1}}=K_{eq,\text{C1}}^{0}\cdot[\text{H}^{\text{+}}]_{\text{x}}^{5}/[\text{H}^{\text{+}}]_{\text{i}}^{4}$, with$K_{eq,C1}^{0}=\exp\left(-\left(\Delta_{r}G_{\text{C1}}^{0}+4F\Delta\Psi\right)/RT\right)$ and $\Delta_{r}G_{\text{C1}}^{0}=\Delta_{f}G_{\text{NAD}}^{0}+\Delta_{f}G_{\text{QH}_{2}}^{0}-\Delta_{r}G_{\text{NADH}}^{0}-\Delta_{r}G_{\text{COQ}}^{0}=-109.7\text{ kJ/mol}$.

Complex III flux:

$J_{\text{C3}}=X_{\text{C3}}\left(\frac{1+[\text{PI}]_{\text{x}}/k_{\text{PI},3}}{1+[\text{PI}]_{\text{x}}/k_{\text{PI},4}}\right)\left(K_{eq,\text{C3}}^{1/2}[\text{Cox}]_{\text{i}}[\text{QH}_{2}]_{\text{x}}^{1/2}-[\text{Cred}]_{\text{i}}[\text{COQ}]_{\text{x}}^{1/2}\right)$,

where $K_{eq,\text{C3}}=K_{eq,\text{C3}}^{0}\cdot[\text{H}^{\text{+}}]_{\text{x}}^{2}/[\text{H}^{\text{+}}]_{\text{i}}^{4}$, with$K_{eq,C3}^{0}=\exp\left(-\left(\Delta_{r}G_{\text{C3}}^{0}+2F\Delta\Psi\right)/RT\right)$ and $\Delta_{r}G_{\text{C3}}^{0}=\Delta_{f}G_{\text{Cred}}^{0}+\Delta_{f}G_{\text{COQ}}^{0}-\Delta_{r}G_{\text{QH2}}^{0}-\Delta_{r}G_{\text{Cox}}^{0}=46.69\text{ kJ/mol}$.

Complex IV flux:

$J_{\text{C4}}=X_{\text{C4}}\left(\frac{1}{1+k_{\text{O}_{2}}/[\text{O}_{2}]}\right)\exp\left(\frac{F\Delta\Psi}{RT}\right)\left(\frac{\text{[Cred]}_{\text{i}}}{\text{cytC}_{\text{tot}}}\right)\left(K_{eq,\text{C4}}^{1/2}[\text{Cred}]_{\text{i}}[\text{O}_{2}]_{\text{x}}^{1/4}-[\text{Cox}]_{\text{i}}\right)$,

where [O_2_]_x_ is assumed to be equal to C_O2,cell_, $K_{eq,\text{C4}}=K_{eq,\text{C4}}^{0}\cdot[\text{H}^{\text{+}}]_{\text{x}}^{4}/[\text{H}^{\text{+}}]_{\text{i}}^{2}$, with $K_{eq,\text{C4}}^{0}=\exp\left(-\left(\Delta_{r}G_{\text{C4}}^{0}+4F\Delta\Psi\right)/RT\right)$ and $\Delta_{r}G_{\text{C4}}^{0}=\Delta_{f}G_{\text{Cox}}^{0}+\Delta_{f}G_{\text{H}_{2}\text{O}}^{0}-\Delta_{r}G_{\text{Cred}}^{0}-\Delta_{r}G_{\text{O}_{2}}^{0}=-202.2\text{ kJ/mol}$.

F_o_F_1_-ATPase flux:

$J_{\text{F1}}=X_{\text{F1}}\left(K_{eq,\text{F1}}[\text{ADP}]_{\text{x}}[\text{PI}]_{\text{x}}-[\text{ATP}]_{\text{x}}\right)$.

where $K_{eq,\text{F1}}=K_{eq,\text{F1}}^{0}\cdot\frac{[\text{H}^{\text{+}}]_{\text{i}}^{n_{\text{A}}}}{[\text{H}^{\text{+}}]_{\text{x}}^{n_{\text{A}}-1}}\cdot\frac{P_{\text{ATP}}}{P_{\text{ADP}}P_{\text{PI}}}$, with$K_{eq,\text{F1}}^{0}=\exp\left(-\left(\Delta_{r}G_{\text{F1}}^{0}-n_{\text{A}}F\Delta\Psi\right)/RT\right)$ and $\Delta_{r}G_{\text{F1}}^{0}=\Delta_{f}G_{\text{ATP}}^{0}+\Delta_{f}G_{\text{H}_{2}\text{O}}^{0}-\Delta_{r}G_{\text{ADP}}^{0}-\Delta_{r}G_{\text{PI}}^{0}=-4.51\text{ kJ/mol}$.

Mitochondrial adenylate kinase flux:

$J_{\text{AKi}}=X_{\text{AKi}}\left(K_{\text{eq,AK}}[\text{ADP}]_{\text{i}}[\text{ADP}]_{\text{i}}-[\text{AMP}]_{\text{i}}[\text{ATP}]_{\text{i}}\right)$.

**Mathematical expressions for TCA cycle fluxes**

For brevity, values of Michaelis, inhibition, or activation constants are not presented here, but provided in Appendix C of [4]. In the following TCA cycle flux expressions, the enzyme activity is represented by *V_mf_*, and *V_mr_* is related to *V_mf_* by obeying the Haledane equation [34].

Pyruvate dehydrogenase flux:

$J_{\text{pdh}}=\frac{V_{mf}\left(1-\frac{1}{K_{eq,\text{pdh}}}\frac{[\text{P}][\text{Q}][\text{R}]}{[\text{A}][\text{B}][\text{C}]}\right)}{K_{mC}\alpha_{i2}[\text{A}][\text{B}]+K_{mB}\alpha_{i1}[\text{A}][\text{C}]+K_{mA}[\text{B}][\text{C}]+[\text{A}][\text{B}][\text{C}]}$,

where [A] = [PYR], [B] = [COASH], [C] = [NAD], [P] = [CO_2_tot], [Q] = [ACCOA], and [R] = [NADH], $K_{eq,\text{pdh}}=K_{eq,\text{pdh}}^{0}\frac{1}{[\text{H}^{\text{+}}]}\frac{P_{\text{CO}_{2}\text{tot}}P_{\text{ACCOA}}P_{\text{NADH}}}{P_{\text{PYR}}P_{\text{COASH}}P_{\text{NAD}}}$ with $K_{eq,\text{pdh}}^{0}=\exp\left(-\frac{\Delta_{r}G_{\text{pdh}}^{0}}{RT}\right)=5.02\times10^{-4}\text{ M}^{\text{-1}}$.

Citrate Synthase flux:

$J_{\text{cits}}=\frac{V_{mf}\left([\text{A}][\text{B}]-\frac{[\text{P}][\text{Q}]}{K_{eq,\text{cits}}}\right)}{K_{ia}K_{mB}\alpha_{i1}+K_{mA}\alpha_{i1}[\text{B}]+K{}_{mB}\alpha_{i2}[\text{A}]+[\text{A}][\text{B}]}$,

where [A] = [OAA], [B] = [ACCOA], [P] = [COASH], and [Q] = [CIT], $K_{eq,\text{cits}}=K_{eq,\text{cits}}^{0}\frac{1}{[\text{H}^{\text{+}}]^{2}}\frac{P_{\text{COA}}P_{\text{CIT}}}{P_{\text{OAA}}P_{\text{ACCOA}}}$ with $K_{eq,\text{cits}}^{0}=\exp\left(-\frac{\Delta_{r}G_{\text{cits}}^{0}}{RT}\right)=7.34\times10^{-8}\text{ M}^{\text{-1}}$.

Aconitase flux:

$J_{\text{acon}}=\frac{V_{mf}V_{mr}\left([\text{A}]-\frac{[\text{P}]}{K_{eq,\text{acon}}}\right)}{K_{mA}V_{mr}+V_{mr}[\text{A}]+\frac{V_{mf}}{K_{eq,\text{acon}}}[\text{P}]}$,

where [A] = [CIT] and [P] = [ICIT], $K_{eq,\text{acon}}=K_{eq,\text{acon}}^{0}\frac{P_{\text{ICIT}}}{P_{\text{CIT}}}$ with $K_{eq,\text{acon}}^{0}=\exp\left(-\frac{\Delta_{r}G_{\text{acon}}^{0}}{RT}\right)=7.59\times10^{-2}$.

Isocitrate dehydrogenase flux:

$J_{\text{isod}}=\frac{V_{mf}\left(1-\frac{1}{K_{eq,\text{isod}}}\frac{[\text{P}][\text{Q}][\text{R}]}{[\text{A}][\text{B}]}\right)}{1+\left(\frac{K_{mB}}{[\text{B}]}\right)^{n_{H}}\alpha_{i}+\frac{K_{mA}}{[\text{A}]}\left(1+\left(\frac{K_{ib}}{[\text{B}]}\right)^{n_{H}}\alpha_{i}+\frac{[\text{Q}]}{K_{iq}}\alpha_{i}\right)}$,

where [A] = [NAD], [B] = [ICIT], [P] = [AKG], [Q] = [NADH], and [R] = [CO_2_tot], $K_{eq,\text{isod}}=K_{eq,\text{isod}}^{0}\frac{1}{[\text{H}^{\text{+}}]^{2}}\frac{P_{\text{AKG}}P_{\text{NADH}}P_{\text{CO}_{2}\text{tot}}}{P_{\text{NAD}}P_{\text{ICIT}}}$ with $K_{eq,\text{isod}}^{0}=\exp\left(-\frac{\Delta_{r}G_{\text{isod}}^{0}}{RT}\right)=3.50\times10^{-16}$.

α-Ketoglutarate dehydrogenase flux:

$J_{\text{akgd}}=\frac{V_{mf}\left(1-\frac{1}{K_{eq,\text{akgd}}}\frac{[\text{P}][\text{Q}][\text{R}]}{[\text{A}][\text{B}][\text{C}]}\right)}{\left(1+\frac{K_{mA}}{[\text{A}]}\alpha_{i}+\frac{K_{mB}}{[\text{B}]}\left(1+\frac{[\text{Q}]}{K_{iq}}\right)+\frac{K_{mC}}{[\text{C}]}\left(1+\frac{[\text{R}]}{K_{ir}}\right)\right)}$,

where [A] = [AKG], [B] = [COASH], [C] = [NAD], [P] = [CO_2_tot], [Q] = [SCOA], and [R] = [NADH], $K_{eq,\text{akgd}}=K_{eq,\text{akgd}}^{0}\frac{1}{[\text{H}^{\text{+}}]}\frac{P_{\text{CO}_{2}\text{tot}}P_{\text{SCOA}}P_{\text{NADH}}}{P_{\text{AKG}}P_{\text{COASH}}P_{\text{NAD}}}$ with $K_{eq,\text{akgd}}^{0}=\exp\left(-\frac{\Delta_{r}G_{\text{akgd}}^{0}}{RT}\right)=6.93\times10^{-3}$.

Succiny-CoA synthetase flux:

$J_{\text{scoas}}=\frac{V_{mf}V_{mr}\left([\text{A}][\text{B}][\text{C}]-\frac{[\text{P}][\text{Q}][\text{R}]}{K_{eq,\text{scoas}}}\right)}{\begin{matrix} V_{mr}K_{ia}K_{ib}K_{mC}+V_{mr}K_{ib}K_{mC}[\text{A}]+V_{mr}K_{ia}K_{mB}[\text{C}] \\ +V_{mr}K_{mC}[\text{A}][\text{B}]+V_{mr}K_{mB}[\text{A}][\text{C}]+V_{mr}K_{mA}[\text{B}][\text{C}]+V_{mr}[\text{A}][\text{B}][\text{C}] \\ +\frac{V_{mf}K_{ir}K_{mQ}[\text{P}]}{K_{eq,\text{scoas}}}+\frac{V_{mf}K_{iq}K_{mP}[\text{R}]}{K_{eq,\text{scoas}}}+\frac{V_{mf}K_{mR}[\text{P}][\text{Q}]}{K_{eq,\text{scoas}}}+\frac{V_{mf}K_{mQ}[\text{P}][\text{R}]}{K_{eq,\text{scoas}}} \\ +\frac{V_{mf}K_{mP}[\text{Q}][\text{R}]}{K_{eq,\text{scoas}}}+\frac{V_{mf}[\text{P}][\text{Q}][\text{R}]}{K_{eq,\text{scoas}}}+\frac{V_{mf}K_{mQ}K_{ir}[\text{A}][\text{P}]}{K_{ia}K_{eq,\text{scoas}}}+\frac{V_{mr}K_{ia}K_{mB}[\text{C}][\text{R}]}{K_{ir}} \\ +\frac{V_{mf}K_{mQ}K_{ir}[\text{A}][\text{B}][\text{P}]}{K_{ia}K_{ib}K_{eq,\text{scoas}}}+\frac{V_{mr}K_{mA}[\text{B}][\text{C}][\text{R}]}{K_{ir}}+\frac{V_{mf}K_{mR}[\text{A}][\text{P}][\text{Q}]}{K_{ia}K_{eq,\text{scoas}}} \\ +\frac{V_{mr}K_{ia}K_{mB}[\text{C}][\text{Q}][\text{R}]}{K_{iq}K_{ir}}+\frac{V_{mf}K_{ir}K_{mQ}[\text{A}][\text{B}][\text{C}][\text{P}]}{K_{ia}K_{ib}K_{ic}K_{eq,\text{scoas}}}+\frac{V_{mf}K_{ip}K_{mR}[\text{A}][\text{B}][\text{C}][\text{Q}]}{K_{ia}K_{ib}K_{ic}K_{eq,\text{scoas}}} \\ +\frac{V_{mf}K_{mR}[\text{A}][\text{B}][\text{P}][\text{Q}]}{K_{ia}K_{ib}K_{eq,\text{scoas}}}+\frac{V_{mr}K_{mA}[\text{B}][\text{C}][\text{Q}][\text{R}]}{K_{iq}K_{ir}}+\frac{V_{mr}K_{mA}K_{ic}[\text{B}][\text{P}][\text{Q}][\text{R}]}{K_{ip}K_{iq}K_{ir}} \\ +\frac{V_{mr}K_{ia}K_{mB}[\text{C}][\text{P}][\text{Q}][\text{R}]}{K_{ip}K_{iq}K_{ir}}+\frac{V_{mf}K_{mR}[\text{A}][\text{B}][\text{C}][\text{P}][\text{Q}]}{K_{ia}K_{ib}K_{ic}K_{eq,\text{scoas}}}+\frac{V_{mr}K_{mA}[\text{B}][\text{C}][\text{P}][\text{Q}][\text{R}]}{K_{ip}K_{iq}K_{ir}} \end{matrix}}$,

where [A] = [GDP], [B] = [SCOA], [C] = [PI], [P] = [COASH], [Q] = [SUC], and [R] = [GTP], $K_{eq,\text{scoas}}=K_{eq,\text{scoas}}^{0}\frac{1}{[\text{H}^{\text{+}}]}\frac{P_{\text{COASH}}P_{\text{SUC}}P_{\text{GTP}}}{P_{\text{GDP}}P_{\text{SCOA}}P_{\text{PI}}}$ with $K_{eq,\text{scoas}}^{0}=\exp\left(-\frac{\Delta_{r}G_{\text{scoas}}^{0}}{RT}\right)=9.54\times10^{\text{-9}}\text{ M}^{\text{-1}}$.

Succinate dehydrogenase flux:

$J_{\text{sdh}}=\frac{V_{mf}V_{mr}\left([\text{A}][\text{B}]-\frac{[\text{P}][\text{Q}]}{K_{eq,\text{sdh}}}\right)}{\begin{matrix} V_{mr}K_{ia}K_{mB}\alpha_{i}+V_{mr}K_{mB}[\text{A}]+V_{mr}K_{mA}\alpha_{i}[\text{B}]+\frac{V_{mf}K_{mQ}\alpha_{i}}{K_{eq,\text{sdh}}}[\text{P}]+\frac{V_{mf}K_{mP}}{K_{eq,\text{sdh}}}[\text{Q}] \\ +V_{mr}[\text{A}][\text{B}]+\frac{V_{mf}K_{mQ}}{K_{eq,\text{sdh}}K_{ia}}[\text{A}][\text{P}]+\frac{V_{mr}K_{mA}}{K_{iq}}[\text{B}][\text{Q}]+\frac{V_{mf}}{K_{eq,\text{sdh}}}[\text{P}][\text{Q}] \end{matrix}}$,

where A] = [SUC], [B] = [COQ], [P] = [QH_2_], and [Q] = [FUM], $K_{eq,\text{sdh}}=K_{eq,\text{sdh}}^{0}\frac{P_{\text{QH}_{2}}P_{\text{FUM}}}{P_{\text{SUC}}P_{\text{COQ}}}$ with $K_{eq,\text{sdh}}^{0}=\exp\left(-\frac{\Delta_{r}G_{\text{sdh}}^{0}}{RT}\right)=1.69$.

Fumarase flux:

$J_{\text{fum}}=\frac{V_{mf}V_{mr}\left([\text{A}]-\frac{[\text{P}]}{K_{eq,\text{fum}}}\right)}{K_{mA}V_{mr}\alpha_{i}+V_{mr}[\text{A}]+\frac{V_{mf}[\text{P}]}{K_{eq,\text{fum}}}}$,

where [A] = [FUM] and [P] = [MAL], $K_{eq,\text{fum}}=K_{eq,\text{fum}}^{0}\frac{P_{\text{MAL}}}{P_{\text{FUM}}}$with $K_{eq,\text{fum}}^{0}=\exp\left(-\frac{\Delta_{r}G_{\text{fum}}^{0}}{RT}\right)=4.04\text{ M}^{\text{-1}}$.

Malate dehydrogenase flux:

$J_{\text{mdh}}=\frac{V_{mf}V_{mr}\left([\text{A}][\text{B}]-\frac{[\text{P}][\text{Q}]}{K_{eq,\text{mdh}}}\right)}{\begin{matrix} V_{mr}K_{ia}K_{mB}\alpha_{i}+V_{mr}K_{mB}[\text{A}]+V_{mr}K_{mA}\alpha_{i}[\text{B}]+\frac{V_{mf}K_{mQ}\alpha_{i}[\text{P}]}{K_{eq,\text{mdh}}}+\frac{V_{mf}K_{mP}[\text{Q}]}{K_{eq,\text{mdh}}} \\ +V_{mr}[\text{A}][\text{B}]+\frac{V_{mf}K_{mQ}[\text{A}][\text{P}]}{K_{eq,\text{mdh}}K_{ia}}+\frac{V_{mf}[\text{P}][\text{Q}]}{K_{eq,\text{mdh}}}+\frac{V_{mr}K_{mA}[\text{B}][\text{Q}]}{K_{iq}} \\ +\frac{V_{mr}[\text{A}][\text{B}][\text{P}]}{K_{ip}}+\frac{V_{mf}[\text{B}][\text{P}][\text{Q}]}{K_{ib}K_{eq,\text{mdh}}} \end{matrix}}$,

where [A] = [NAD], [B] = [MAL], [P] = [OAA], and [Q] = [NADH], $K_{eq,\text{mdh}}=K_{eq,\text{mdh}}^{0}\frac{1}{[\text{H}^{\text{+}}]}\frac{P_{\text{OAA}}P_{\text{NADH}}}{P_{\text{NAD}}P_{\text{MAL}}}$ with $K_{eq,\text{mdh}}^{0}=\exp\left(-\frac{\Delta_{r}G_{\text{mdh}}^{0}}{RT}\right)=2.27\times10^{-12}$.

Nucleoside diphosphokinase flux:

$J_{\text{ndk}}=\frac{V_{mf}V_{mr}\left([\text{A}][\text{B}]-\frac{[\text{P}][\text{Q}]}{K_{eq,\text{ndk}}}\right)/\alpha_{i}}{\begin{matrix} V_{mr}K_{mB}[\text{A}]+V_{mr}K_{mA}[\text{B}]+\frac{V_{mf}K_{mQ}[\text{P}]}{K_{eq,\text{ndk}}}+\frac{V_{mf}K_{mP}[\text{Q}]}{K_{eq,\text{ndk}}}+V_{mr}[\text{A}][\text{B}] \\ +\frac{V_{mf}K_{mQ}[\text{A}][\text{P}]}{K_{eq,\text{ndk}}K_{ia}}+\frac{V_{mf}[\text{P}][\text{Q}]}{K_{eq,\text{ndk}}}+\frac{V_{mr}K_{mA}[\text{B}][\text{Q}]}{K_{iq}} \end{matrix}}$,

where [A] = [GTP], [B] = [ADP], [P] = [GDP], and [Q] = [ATP], $K_{eq,\text{nkd}}=K_{eq,\text{nkd}}^{0}$with $K_{eq,\text{ndk}}^{0}=\exp\left(-\frac{\Delta_{r}G_{\text{ndk}}^{0}}{RT}\right)=1$.

Glutamate oxaloacetate transaminase flux:

$J_{\text{got}}=\frac{V_{mf}V_{mr}\left([\text{A}][\text{B}]-\frac{[\text{P}][\text{Q}]}{K_{eq,\text{got}}}\right)}{\begin{matrix} V_{mr}K_{mB}[\text{A}]+V_{mr}K_{mA}[\text{B}]+\frac{V_{mf}K_{mQ}[\text{P}]}{K_{eq,\text{got}}}+\frac{V_{mf}K_{mP}[\text{Q}]}{K_{eq,\text{got}}}+V_{mr}[\text{A}][\text{B}] \\ +\frac{V_{mf}K_{mQ}[\text{A}][\text{P}]}{K_{eq,\text{got}}K_{ia}}+\frac{V_{mf}[\text{P}][\text{Q}]}{K_{eq,\text{got}}}+\frac{V_{mr}K_{mA}[\text{B}][\text{Q}]}{K_{iq}} \end{matrix}}$,

where [A] = [ASP], [B] = [AKG], [P] = [OAA], and [Q] = [GLU], $K_{eq,\text{got}}=K_{eq,\text{got}}^{0}\frac{P_{\text{OAA}}P_{\text{GLU}}}{P_{\text{ASP}}P_{\text{AKG}}}$ with $K_{eq,\text{got}}^{0}=\exp\left(-\frac{\Delta_{r}G_{\text{got}}^{0}}{RT}\right)=1.77$.

**Mathematical expressions for substrate and cation transport across the inner mitochondrial membrane**

Adenine nucleotide translocase (ANT) flux:

$J_{\text{ANT}}=x_{\text{ANT}}\frac{k_{2}^{\text{ANT}}q\frac{[\text{ATP}^{\text{4-}}]_{\text{x}}[\text{ADP}^{\text{3-}}]_{\text{i}}}{K_{o}^{\text{D}}}-k_{3}^{\text{ANT}}\frac{[\text{ATP}^{\text{4-}}]_{\text{i}}[\text{ADP}^{\text{3-}}]_{\text{x}}}{K_{o}^{\text{T}}}}{\left(1+\frac{[\text{ATP}^{\text{4-}}]_{\text{i}}}{K_{o}^{\text{T}}}+\frac{[\text{ADP}^{\text{3-}}]_{\text{i}}}{K_{o}^{\text{D}}}\right)\left(\frac{[\text{ATP}^{\text{4-}}]_{\text{x}}}{K_{o}^{\text{T}}}+q\frac{[\text{ADP}^{\text{3-}}]_{\text{x}}}{K_{o}^{\text{D}}}\right)}$,

where

$k_{2}^{\text{ANT}}=k_{2}^{\text{ANT,o}}\exp\left(\left(-3a_{1}-4a_{2}+a_{3}\right)\frac{F\Delta\Psi}{RT}\right)$,

$k_{3}^{\text{ANT}}=k_{3}^{\text{ANT,o}}\exp\left(\left(-4a_{1}-3a_{2}+a_{3}\right)\frac{F\Delta\Psi}{RT}\right)$,

$K_{o}^{\text{D}}=K_{o}^{\text{D,o}}\exp\left(\frac{3\delta_{D}RT}{F\Delta\Psi}\right)$,

$K_{o}^{\text{T}}=K_{o}^{\text{T,o}}\exp\left(\frac{4\delta_{T}RT}{F\Delta\Psi}\right)$,

and

$q=\frac{k_{3}^{\text{ANT}}K_{o}^{\text{D}}}{k_{2}^{\text{ANT}}K_{o}^{\text{T}}}\exp\left(\frac{F\Delta\Psi}{RT}\right)$,

with $k_{2}^{\text{ANT,o}}$= 0.159 sec^-1^, $k_{3}^{\text{ANT,o}}$= 0.501 sec^-1^, $K_{o}^{\text{D},o}$= 38.89 mM, $K_{o}^{\text{T},o}$= 56.05 mM, *a*_1_ = 0.2829, *a*_2_ = -0.2086, *a*_3_ = 0.2372, *δ*_T_ = 0.0167, and *δ*_D_ = 0.0699 (cited from Supplemental Material of [1]).

Phosphate-hydrogen co-transporter flux:

$J_{\text{PIHt}}=X_{\text{PIHt}}\cdot\frac{[\text{H}_{2}\text{PO}_{4}^{\text{-}}]_{\text{i}}[\text{H}^{\text{+}}]_{\text{i}}-[\text{H}_{2}\text{PO}_{4}^{\text{-}}]_{\text{x}}[\text{H}^{\text{+}}]_{\text{x}}}{k_{\text{PIHt}}\left(1+[\text{H}_{2}\text{PO}_{4}^{\text{-}}\text{]}_{\text{i}}\text{/}k_{\text{PIHt}}\right)\left(1+[\text{H}_{2}\text{PO}_{4}^{\text{-}}\text{]}_{\text{x}}\text{/}k_{\text{PIHt}}\right)}$.

Potassium-hydrogen exchange flux:

$J_{\text{KH}}=X_{\text{KH}}\left([\text{K}^{\text{+}}]_{\text{i}}[\text{H}^{\text{+}}]_{\text{x}}-[\text{K}^{\text{+}}]_{\text{x}}[\text{H}^{\text{+}}]_{\text{i}}\right)$.

Pyruvate-hydrogen co-transporter flux:

$J_{\text{PYRH}}=X_{\text{PYRH}}\left([\text{PYR}^{\text{-}}]_{\text{i}}[\text{H}^{\text{+}}]_{\text{i}}-[\text{PYR}^{\text{-}}]_{\text{x}}[\text{H}^{\text{+}}]_{\text{x}}\right)$.

Glutamate-hydrogen co-transporter flux:

$J_{\text{GLUH}}=X_{\text{GLUH}}\left([\text{GLU}^{\text{-}}]_{\text{i}}[\text{H}^{\text{+}}]_{\text{i}}-[\text{GLU}^{\text{-}}]_{\text{x}}[\text{H}^{\text{+}}]_{\text{x}}\right)$.

Citrate-malate exchange flux:

$J_{\text{CITMAL}}=X_{\text{CITMAL}}\left([\text{HCIT}^{\text{2-}}]_{\text{i}}[\text{MAL}^{\text{2-}}]_{\text{x}}-[\text{HCIT}^{\text{2-}}]_{\text{x}}[\text{MAL}^{\text{2-}}]_{\text{i}}\right)$.

α-Ketoglutarate-malate exchange flux:

$J_{\text{AKGMAL}}=\frac{X_{\text{AKGMAL}}\left([\text{AKG}^{\text{2-}}]_{\text{i}}[\text{MAL}^{\text{2-}}]_{\text{x}}-[\text{AKG}^{\text{2-}}]_{\text{x}}[\text{MAL}^{\text{2-}}]_{\text{i}}\right)}{\begin{matrix} r_{\text{AKGMAL}}\alpha_{out1}+(1-r_{\text{AKGMAL}})\alpha_{in1}+[\text{AKG}^{\text{2-}}]_{\text{i}}[\text{MAL}^{\text{2-}}]_{\text{x}}\alpha_{in2}/K_{d,\text{AKGMAL}} \\ +[\text{AKG}^{\text{2-}}]_{\text{x}}[\text{MAL}^{\text{2-}}]_{\text{i}}\alpha_{out2}/K_{d,\text{AKGMAL}} \\ +[\text{AKG}^{\text{2-}}]_{\text{i}}[\text{MAL}^{\text{2-}}]_{\text{x}}[\text{AKG}^{\text{2-}}]_{\text{x}}[\text{MAL}^{\text{2-}}]_{\text{i}}/K_{d,\text{AKGMAL}}^{2} \end{matrix}}$,

where *r*_AKGMAL_ and *K_d_,*_AKGMAL_ are adjustable parameters, and *α_out1_*, *α_in1_*, *α_out2_*, and *α_in2_* are inhibition coefficients for the exchanger. The inhibition coefficients are computed as:

$$\alpha_{out1}=1+[\text{CIT}]_{\text{i}}/K_{i\text{CIT}}+[\text{GLU}]_{\text{i}}/K_{i\text{GLU}}+[\text{ASP}]_{\text{i}}/K_{i\text{ASP}}+[\text{SUC}]_{\text{i}}/K_{i\text{SUC}},$$

$$\alpha_{in1}=1+[\text{CIT}]_{\text{x}}/K_{i\text{CIT}}+[\text{GLU}]_{\text{x}}/K_{i\text{GLU}}+[\text{ASP}]_{\text{x}}/K_{i\text{ASP}}+[\text{SUC}]_{\text{x}}/K_{i\text{SUC}},$$

$$\alpha_{out2}=1+r_{\text{AKGMAL}}\left([\text{CIT}]_{\text{i}}/K_{i\text{CIT}}+[\text{GLU}]_{\text{i}}/K_{i\text{GLU}}+[\text{ASP}]_{\text{i}}/K_{i\text{ASP}}+[\text{SUC}]_{\text{i}}/K_{i\text{SUC}}\right),$$

$$\alpha_{in2}=1+r_{\text{AKGMAL}}\left([\text{CIT}]_{\text{x}}/K_{i\text{CIT}}+[\text{GLU}]_{\text{x}}/K_{i\text{GLU}}+[\text{ASP}]_{\text{x}}/K_{i\text{ASP}}+[\text{SUC}]_{\text{x}}/K_{i\text{SUC}}\right),$$

with *K_i_*_CIT_ = 3.6 mM, *K_i_*_GLU_ = 2.5 mM, *K_i_*_ASP_ = 2.7 mM, and *K_i_*_SUC_ = 1.6 mM (cited from reference [35]).

Succinate/phosphate exchange flux:

$J_{\text{SUCPI}}=X_{\text{SUCPI}}\left([\text{SUC}^{\text{2-}}\text{]}_{\text{i}}[\text{PI}^{\text{2-}}]_{\text{x}}-[\text{SUC}^{\text{2-}}]_{\text{x}}[\text{PI}^{\text{2-}}]_{\text{i}}\right)$.

Malate/phosphate exchange flux:

$J_{\text{MALPI}}=X_{\text{MALPI}}\left([\text{MAL}^{\text{2-}}]_{\text{i}}[\text{PI}^{\text{2-}}]_{\text{x}}-[\text{MAL}^{\text{2-}}]_{\text{x}}[\text{PI}^{\text{2-}}]_{\text{i}}\right)$.

Fumarate-succinate exchange flux:

$J_{\text{FUMSUC}}=X_{\text{FUMSUC}}\left([\text{FUM}^{\text{2-}}\text{]}_{\text{i}}[\text{SUC}^{\text{2-}}]_{\text{x}}-[\text{FUM}^{\text{2-}}]_{\text{x}}[\text{SUC}^{\text{2-}}]_{\text{i}}\right)$.

Aspartate-glutamate exchange flux:

$J_{\text{ASPGLU}}=X_{\text{ASPGLU}}\cdot\frac{e^{-F\Delta\Psi/RT}[\text{ASP}^{\text{-}}]_{\text{i}}[\text{HGLU}^{0}]_{\text{x}}-[\text{ASP}^{\text{-}}]_{\text{x}}[\text{HGLU}^{0}]_{\text{i}}}{\left(1+[\text{ASP}^{\text{-}}]_{\text{x}}[\text{HGLU}^{0}]_{\text{i}}/K_{d,\text{ASPGLU}}\right)\left(1+[\text{ASP}^{\text{-}}]_{\text{i}}[\text{HGLU}^{0}]_{\text{x}}/K_{d,\text{ASPGLU}}\right)}$,

where *K_d_,*_ASPGLU_ is an adjustable parameter.

Proton leak flux:

$J_{\text{Hle}}=X_{\text{Hle}}\Delta\Psi\left(\frac{[\text{H}^{\text{+}}]_{\text{c}}e^{+F\Delta\Psi/RT}-[\text{H}^{\text{+}}]_{\text{x}}}{e^{+F\Delta\Psi/RT}-1}\right)$.

**Mathematical expressions for passive permeation across the outer mitochondrial membrane**

Adenine nucleoside permeation fluxes:

$$J_{\text{ATPt}}=\gamma p_{A}\left([\text{ATP}]_{\text{c}}-[\text{ATP}]_{\text{i}}\right)$$

$$J_{\text{ADPt}}=\gamma p_{A}\left([\text{ADP}]_{\text{c}}-[\text{ADP}]_{\text{i}}\right)$$

$J_{\text{AMPt}}=\gamma p_{A}\left([\text{AMP}]_{\text{c}}-[\text{AMP}]_{\text{i}}\right)$.

Inorganic phosphate permeation flux:

$J_{\text{PIt}}=\gamma p_{\text{PI}}\left([\text{PI}]_{\text{c}}-[\text{PI}]_{\text{i}}\right)$.

TCA cycle intermediate permeation fluxes:

$$J_{\text{PYRt}}=\gamma p_{\text{TI}}\left([\text{PYR}]_{\text{c}}-[\text{PYR]}_{\text{i}}\right)$$

$$J_{\text{CITt}}=\gamma p_{\text{TI}}\left([\text{CIT}]_{\text{c}}-[\text{CIT}]_{\text{i}}\right)$$

$$J_{\text{MALt}}=\gamma p_{\text{TI}}\left([\text{MAL}]_{\text{c}}-[\text{MAL}]_{\text{i}}\right)$$

$$J_{\text{AKGt}}=\gamma p_{\text{TI}}\left([\text{AKG}]_{\text{c}}-[\text{AKG}]_{\text{i}}\right)$$

$$J_{\text{SUCt}}=\gamma p_{\text{TI}}\left([\text{SUC}]_{\text{c}}-[\text{SUC}]_{\text{i}}\right)$$

$$J_{\text{FUMt}}=\gamma p_{\text{TI}}\left([\text{FUM}]_{\text{c}}-[\text{FUM}]_{\text{i}}\right)$$

$$J_{\text{GLUt}}=\gamma p_{\text{TI}}\left([\text{GLU}]_{\text{c}}-[\text{GLU}]_{\text{i}}\right)$$

$J_{\text{ASPt}}=\gamma p_{\text{TI}}\left([\text{ASP}]_{\text{c}}-[\text{ASP}]_{\text{i}}\right)$.

**Mathematical expressions for cytoplasmic reaction fluxes**

Mitochondrial adenylate kinase flux:

$J_{\text{AKi}}=X_{\text{AKi}}\left(K_{\text{eq,AK}}[\text{ADP}^{\text{3-}}]_{\text{i}}^{2}-[\text{AMP}^{\text{2-}}]_{\text{i}}[\text{ATP}^{\text{4-}}]_{\text{i}}\right)$,

where *X*_AKi_ is an large arbitrary value to maintain the reaction around equilibrium.

Cytoplasmic adenylate kinase flux:

$J_{\text{AKc}}=X_{\text{AKc}}\left(K_{\text{eq,AK}}[\text{ADP}^{\text{3-}}]_{\text{c}}^{2}-[\text{AMP}^{\text{2-}}]_{\text{c}}[\text{ATP}^{\text{4-}}]_{\text{c}}\right)$,

where *X*_AKc_ is an large arbitrary value to maintain the reaction around equilibrium.

Creatine kinase flux:

$J_{\text{CKc}}=X_{\text{CKc}}\left(K_{\text{eq,CK}}[\text{ADP}^{\text{3-}}]_{\text{c}}[\text{PCr}^{\text{2-}}]_{\text{c}}[\text{H}^{\text{+}}]_{\text{c}}-[\text{ATP}^{\text{4-}}]_{\text{c}}[\text{Cr}^{0}]_{\text{c}}\right)$,

where *X*_CKc_ is an large arbitrary value to maintain the reaction around equilibrium.

# References

1. Wu F, Zhang EY, Zhang J, Bache RJ, Beard DA: **Phosphate metabolite concentrations and ATP hydrolysis potential in normal and ischemic hearts.** *J Physiol* 2008, **586:**4193-4208.

2. Beard DA: **Modeling of oxygen transport and cellular energetics explains observations on in vivo cardiac energy metabolism.** *PLoS Comput Biol* 2006, **2:**e107.

3. Beard DA, Vinnakota KC, Wu F: **Detailed enzyme kinetics in terms of biochemical species: study of citrate synthase.** *PLoS ONE* 2008, **3:**e1825.

4. Wu F, Yang F, Vinnakota KC, Beard DA: **Computer modeling of mitochondrial tricarboxylic acid cycle, oxidative phosphorylation, metabolite transport, and electrophysiology.** *J Biol Chem* 2007, **282:**24525-24537.

5. Altman PL, Dittmer DS: *Respiration and circulation.* Bethesda, Md.,: Federation of American Societies for Experimental Biology; 1971.

6. Christoforides C, Laasberg LH, Hedley-Whyte J: **Effect of temperature on solubility of O2 in human plasma.** *J Appl Physiol* 1969, **26:**56-60.

7. Mahler M, Louy C, Homsher E, Peskoff A: **Reappraisal of diffusion, solubility, and consumption of oxygen in frog skeletal muscle, with applications to muscle energy balance.** *J Gen Physiol* 1985, **86:**105-134.

8. Hellums JD, Nair PK, Huang NS, Ohshima N: **Simulation of intraluminal gas transport processes in the microcirculation.** *Ann Biomed Eng* 1996, **24:**1-24.

9. McGuire BJ, Secomb TW: **A theoretical model for oxygen transport in skeletal muscle under conditions of high oxygen demand.** *J Appl Physiol* 2001, **91:**2255-2265.

10. Beard DA, Bassingthwaighte JB: **Advection and diffusion of substances in biological tissues with complex vascular networks.** *Ann Biomed Eng* 2000, **28:**253-268.

11. Pagel PS, Hettrick DA, Montgomery MW, Kersten JR, Steffen RP, Warltier DC: **RSR13, a synthetic modifier of hemoglobin-oxygen affinity, enhances the recovery of stunned myocardium in anesthetized dogs.** *J Pharmacol Exp Ther* 1998, **285:**1-8.

12. Schenkman KA, Marble DR, Burns DH, Feigl EO: **Myoglobin oxygen dissociation by multiwavelength spectroscopy.** *J Appl Physiol* 1997, **82:**86-92.

13. Vinnakota KC, Bassingthwaighte JB: **Myocardial density and composition: a basis for calculating intracellular metabolite concentrations.** *Am J Physiol Heart Circ Physiol* 2004, **286:**H1742-1749.

14. Kassab GS, Fung YC: **Topology and dimensions of pig coronary capillary network.** *Am J Physiol* 1994, **267:**H319-325.

15. Caldwell JH, Martin GV, Raymond GM, Bassingthwaighte JB: **Regional myocardial flow and capillary permeability-surface area products are nearly proportional.** *Am J Physiol* 1994, **267:**H654-666.

16. Deussen A, Bassingthwaighte JB: **Modeling [15O]oxygen tracer data for estimating oxygen consumption.** *Am J Physiol* 1996, **270:**H1115-1130.

17. Wu F, Jeneson JA, Beard DA: **Oxidative ATP synthesis in skeletal muscle is controlled by substrate feedback.** *Am J Physiol Cell Physiol* 2007, **292:**C115-124.

18. Vogt M, Puntschart A, Geiser J, Zuleger C, Billeter R, Hoppeler H: **Molecular adaptations in human skeletal muscle to endurance training under simulated hypoxic conditions.** *J Appl Physiol* 2001, **91:**173-182.

19. Beard DA: **A Biophysical Model of the Mitochondrial Respiratory System and Oxidative Phosphorylation.** *PLoS Comput Biol* 2005, **1:**e36.

20. Munoz DR, de Almeida M, Lopes EA, Iwamura ES: **Potential definition of the time of death from autolytic myocardial cells: a morphometric study.** *Forensic Sci Int* 1999, **104:**81-89.

21. Tomashek JJ, Brusilow WS: **Stoichiometry of Energy Coupling by Proton-Translocating ATPases: A History of Variability.** *J Bioenerg Biomembr* 2000, **32:**493-500.

22. Vendelin M, Kongas O, Saks V: **Regulation of mitochondrial respiration in heart cells analyzed by reaction-diffusion model of energy transfer.** *American Journal of Physiology - Cell Physiology* 2000, **278:**C747-764.

23. Lee AC, Zizi M, Colombini M: **Beta-NADH decreases the permeability of the mitochondrial outer membrane to ADP by a factor of 6.** *J Biol Chem* 1994, **269:**30974-30980.

24. Gentet LJ, Stuart GJ, Clements JD: **Direct measurement of specific membrane capacitance in neurons.** *Biophys J* 2000, **79:**314-320.

25. Kapus A, Ligeti E, Fonyo A: **Na+/H+ exchange in mitochondria as monitored by BCECF fluorescence.** *FEBS Lett* 1989, **251:**49-52.

26. Vaughan-Jones RD, Peercy BE, Keener JP, Spitzer KW: **Intrinsic H(+) ion mobility in the rabbit ventricular myocyte.** *J Physiol* 2002, **541:**139-158.

27. Jeneson JA, Westerhoff HV, Brown TR, Van Echteld CJ, Berger R: **Quasi-linear relationship between Gibbs free energy of ATP hydrolysis and power output in human forearm muscle.** *Am J Physiol* 1995, **268:**C1474-1484.

28. Veech RL, Lawson JW, Cornell NW, Krebs HA: **Cytosolic phosphorylation potential.** *J Biol Chem* 1979, **254:**6538-6547.

29. Alberty RA: *Thermodynamics of Biochemical Reactions.* Hoboken, N.J.: John Wiley & Sons; 2003.

30. NIST: **Database 46: Critical Stability Constants.** National Institute of Standards and Technology.

31. Wu F, Jeneson JAL, Beard DA: **Oxidative ATP synthesis in skeletal muscle is controlled by substrate feedback.** *Am J Physiol--Cell Physiol* 2006**:**(in press).

32. Beard DA: **Modeling of Oxygen Transport and Cellular Energetics Explains Observations on In Vivo Cardiac Energy Metabolism.** *PLoS Comput Biol* 2006, **2**.

33. Beard DA, Qian H: *Chemical Biophysics: Quantitative Analysis of Cellular Systems* 1edn: Cambridge University Press; 2007.

34. Segel IH: *Enzyme Kinetics.* New York: Wiley Interscience; 1975.

35. Palmieri F, Quagliariello E, Klingenberger M: **Kinetics and specificity of the oxoglutarate carrier in rat-liver mitochondria.** *Eur J Biochem* 1972, **29:**408-416.
